# In Vitro Pro-Apoptotic and Anti-Migratory Effects of *Marantodes pumilum* (syn. *Labisia pumila*) Extracts on Human Prostate Cancer Cell Lines: Bioguided Isolation of 5-Henicosene-1-yl-resorcinol

**DOI:** 10.3390/plants12071576

**Published:** 2023-04-06

**Authors:** Mohd Mukrish Mohd Hanafi, Harisun Yaakob, Simon Gibbons, Jose M. Prieto

**Affiliations:** 1 Department of Pharmaceutical and Biological Chemistry, U.C.L. School of Pharmacy, London WC1N 1AX, UK; mohd.hanafi.12@ucl.ac.uk (M.M.M.H.); s.gibbons@ljmu.ac.uk (S.G.); 2 Institute of Bioproduct Development (IBD), Universiti Teknologi Malaysia, Johor Bahru 81310, Malaysia; harisun@utm.my; 3 School of Pharmacy, University of East Anglia, Norwich NR4 7TJ, UK; 4 School of Pharmacy and Biomolecular Sciences, Liverpool John Moores University, Liverpool L3 3AF, UK

**Keywords:** apoptosis, migration, invasion, prostate cancer, *Marantodes pumilum*

## Abstract

This study aims to evaluate the in vitro cytotoxic and anti-migratory effects of *Marantodes pumilum* Blume Kuntze plant extracts on prostate cancer cells, identify the active compound/s, and characterize their mechanism of action. The crude methanolic extract was partitioned into *n*-hexane (MPh), chloroform (MPc), and aqueous (MPa) extracts. Antiproliferative fractions (IC50 < 30 μg/mL based on SRB staining of LNCaP and PC3 cell lines) were further fractionated. Active compound/s were identified using spectroscopic methods. In vitro mechanistic studies on PC3 cells included: annexin V-FITC staining, mitochondrial membrane potential (MMP) depolarization measurements, the activity of caspases 3 and 7, nuclear DNA fragmentation, cell cycle analysis, modulation of Bax, Bcl-2, Smac/Diablo, Alox-5, VEGF-A, CXCR4, and CXCL12 mRNA gene expression via RT-PCR, 2D migration (scratch assay), and 3D invasion (Boyden chamber). MPc extract was the most active, inducing cell death (*p <* 0.05) via apoptosis, as evidenced by nuclear DNA fragmentation and an increase in MMP depolarization (*p <* 0.05) as well as the activation of caspases 3/7 (MPc *p <* 0.01) in both PC3 and LNCaP cell lines. In addition, MPc upregulated Bax and Smac/DIABLO, downregulated Bcl-2 (*p <* 0.05), and inhibited ALOX-5 mRNA gene expression (*p <* 0.001). MPc was not cytotoxic against normal human fibroblast cells (HDFa) at the tested concentrations. Moreover, MPc inhibited migration and invasion of PC3 cells (*p <* 0.01). These effects were accompanied by the downregulation of both VEGF-A and CXCL-12 gene expressions (*p <* 0.001). A monounsaturated 5-alkyl resorcinol was isolated as the active compound in the MPc extract and identified as 5-henicosene-1-yl-resorcinol.

## 1. Introduction

Prostate cancer is a significant global health problem and a scientific challenge accounting for 6.6% of the deaths from cancer in men [1]. Although the incidence of prostate cancer is more prevalent in Western countries, the number of prostate cancer cases has also snowballed in Asian countries such as Japan [2,3]. According to the statistics, prostate cancer ranked ninth overall and is the fourth most frequent cancer (7.3% of all cancers) diagnosed in Malaysian men [4].

Many cases of prostate cancer could be prevented, and phytochemicals may be used as chemopreventive agents if integrated into nutritional interventions [5]. Malaysia is interested in natural remedies, and its government is exploring the potential of its diverse plant life, including 1200 medicinal plants, to become newer, more sustainable sources of food and medicines [6]. In a recent review, we describe the potential of the top ten Malay plants prioritized by the Malaysian government, including, *Marantodes pumilum* (syn. *Labisia pumila*) [7]. Preliminary research on this economically and culturally important Malay medicinal plant revealed promising cytotoxicity of the extracts and some of its phytochemicals [8].

This research investigates the pro-apoptotic activities of *Marantodes pumilum* (Blume) Kuntze (synonym *Labisia pumila* var *pumila*) and characterizes for the first time the main cellular mechanisms of its cytotoxic properties. In addition, we also explore the involvement of resistance factors such as Smac/DIABLO and the role of the CXCL12/CXCR4 axis in survival and migration/invasion, respectively.

## 2. Results

### 2.1. Plant Extracts

The yields of each extract were: MPc = 0.42%, MPh = 0.11%, and MPa = 3.44%.

The HPLC-DAD-UV fingerprint for each extract is presented in Figures S1–S3 (Supplementary Materials).

### 2.2. Cell Viability

The MPc extract was the most cytotoxic against the selected prostate cancer cell lines, PC3, DU145, and LNCaP, with an IC50 value of less than 15 μg/mL (Table 1). On the other hand, paclitaxel was a reference drug and gave similar IC50 (≤0.01 μM) in all lines. Therefore, according to Suffness and Pezzuto [9], extracts with an IC50 value of less than 30 μg/mL are to be considered promising and selected for further bioguided fractionation.

### 2.3. Cell Morphology

In this study, the morphological changes in the prostate cancer cell lines (LNCaP and PC3) untreated and treated with the active extracts of *Marantodes pumilum* were observed using an EVOS^®^F.L. Imaging System at 24, 48, and 72 h. The characteristics of apoptosis, such as cells detachment from the substratum, cell shrinkage, nuclear condensation, membrane blebbing, and the formation of apoptotic bodies, were detected in the treated cells. In addition, the reduction in the cell population was evident when comparing the untreated and the treated cells (Figures S4–S6, Supplementary Materials).

### 2.4. Pro-Apoptotic Effect of the Active Extracts of Marantodes pumilum (MP)

#### 2.4.1. Stimulation of Caspases 3/7 Activity

**Figure 1:**
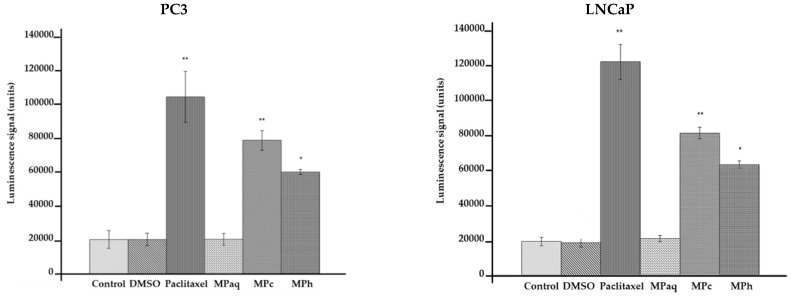
Caspase 3/7 activity in PC3 and LNCaP cells treated with the active extracts of *Marantodes pumilum* for 72 h. The y-axis shows the luminescent signal subtracted from a blank and is proportional to the activity of caspases. The error bars display the standard error of the mean (SEM) obtained from 3 independent experiments. Significance compared to control, * (*p <* 0.05) and ** (*p <* 0.01), as determined via unpaired *t*-test.

Both LNCaP and PC3 (Figure 1) cells treated with either MPc or MPh extracts showed a significant increase (*p <* 0.01 and *p <* 0.05, respectively) in the activity of caspases 3 and 7 when compared to the untreated cells.

#### 2.4.2. Detection of Apoptotic Cells Using Annexin-V FITC Staining

**Figure 2:**
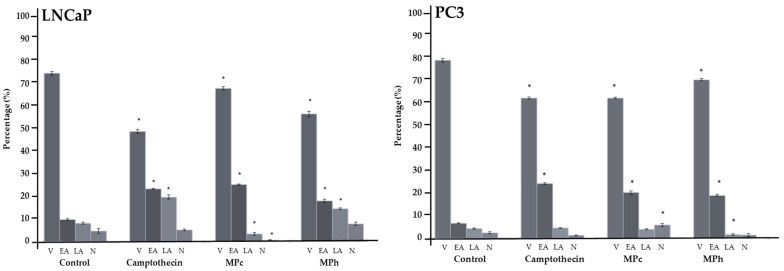
Flow cell cytometry analyses using Annexin V-FITC and PI staining of LNCaP and PC3 cells after 6 h of treatment with the cytotoxic extracts of *Marantodes pumilum* or camptothecin (positive control). V = viable cells; EA = early apoptotic cells; LA = late or secondary apoptotic cells; N = necrotic cells. The error bars display the standard error of the mean (SEM) obtained from 3 independent experiments. Significance compared to control, * (*p <* 0.05) as determined via unpaired *t*-test.

Results in Figure 2 show that there was a significant increase (*p <* 0.05) in the population of LNCaP cells in the early apoptosis phase after the treatment with MPc when compared to the untreated cells. LNCaP cells treated with MPh also show a significant increase (*p <* 0.05) in early apoptosis. PC3 cells treated with MPc showed a similar trend (*p <* 0.05) in the cell population in the early apoptosis phase when compared to the untreated cells. Treatment with MPh caused a significant increase (*p <* 0.05) in the cell population in the early apoptosis phase when compared to the untreated cells.

#### 2.4.3. Cell Cycle Analyses

**Figure 3:**
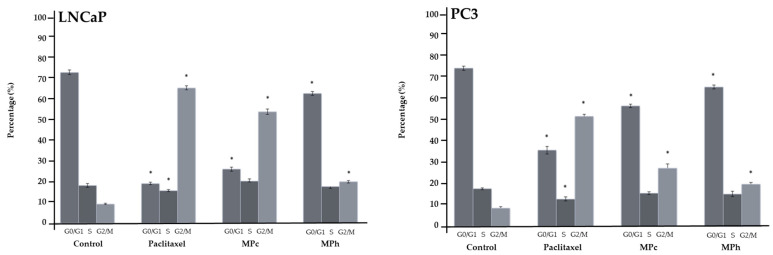
Cell cycle distribution of cell populations (%). LNCaP and PC3 cells were treated for 48 h with the IC50 of the cytotoxic extracts or paclitaxel (positive control). The results shown are representatives of three independent experiments. The error bars display the standard error of the mean (SEM) obtained from 3 independent experiments. Significance compared to control, * (*p <* 0.05) as determined via unpaired *t*-test.

There was a significant increase (*p* < 0.05) in the population of LNCaP cells at the G2M phase as compared to the untreated cells when treated with the IC50 of both MPc and MPh extracts (Figure 3). The same trend was observed when the PC3 cells were treated with the IC50 of both MPc and MPh. In addition, treatment with the IC50 of both plant extracts caused a significant (*p <* 0.05) decrease in the LNCaP and PC3 cells’ population at the G0/G1 phase, while the percentages of cell population at the S phase remained almost the same for both cell lines.

### 2.5. Further Mechanistic Studies of the Cytotoxic Effect of M. pumilum Extracts in PC3 Cells

#### 2.5.1. Nuclear DNA Fragmentation

**Figure 4:**
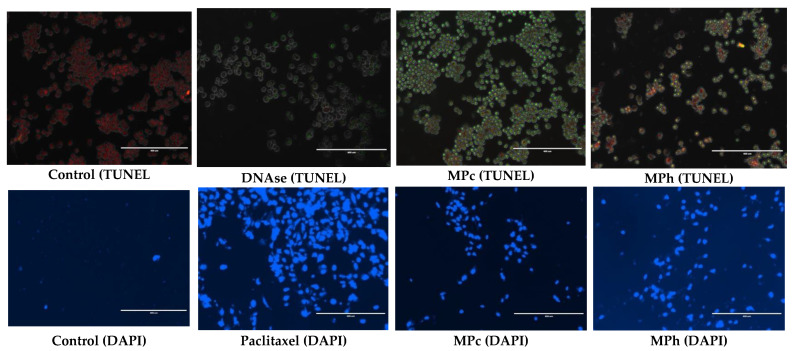
PC3 cells were treated with MPc and MPh extracts and DNAse (positive control) and stained with either Dead-End™ Fluorometric TUNEL or DAPI after 72 h of treatment. TUNEL: red fluorescence indicated healthy cells, while green fluorescence showed fragmented nuclear DNA. DAPI: blue fluorescence indicates nuclear (chromatin) condensation. Images are representative of 3 independent experiments.

After the previous results, we decided to focus on PC3 cells to find out the mechanism of action of the extracts. Figure 4 shows that after 72 h of treatment with active plant extracts, apoptotic cells show localized green fluorescence (fluorescein-12-dUTP).

#### 2.5.2. Modulation of Apoptotic-Related mRNA Gene Expression

**Figure 5:**
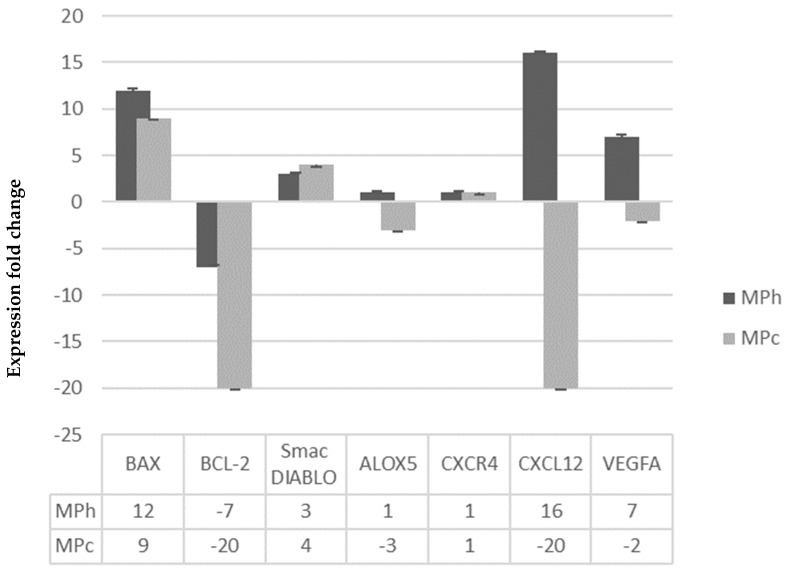
Modulation of mRNA gene expression in PC3 cells treated with MNTC of the active plant extracts of *Marantodes pumilum* after 96 h. The expression fold change was determined as described in RT-qPCR conditions and analysis. Data are means ± SD; *n* = 4.

Results in Figure 5 show that MPc was able to increase the expression of Bax and Smac/DIABLO, respectively (*p <* 0.001), while reducing the expression of Bcl-2 in a significant manner. MPh also significantly stimulates the expression of both Bax and Smac/DIABLO (*p <* 0.05) while inhibiting Bcl-2 expression (*p <* 0.05), respectively. These findings suggest that both MPc and MPh increase the expression of pro-apoptotic genes and reduce the expression of anti-apoptotic genes, which could lead to the increased production of pro-apoptotic proteins and reduced production of anti-apoptotic proteins and ultimately induce PC3 cells death via apoptosis.

#### 2.5.3. Stimulation of Apoptosis through Inactivation of ALOX-5 mRNA Gene Expression

Figure 5 shows that only MPc could cause significant inactivation to the mRNA gene expression of ALOX-5 (*p <* 0.001). This suggests that MPc may also trigger PC3 cell death via apoptosis by inhibiting ALOX-5 gene expression in addition to activating the intrinsic apoptosis pathway [10].

#### 2.5.4. Mitochondrial Membrane Potential Depolarization in PC3 Cell Line Treated with the Active Plant Extracts

**Figure 6:**
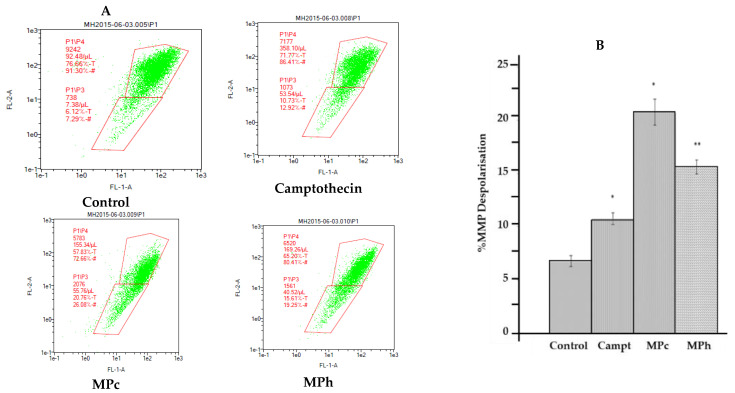
Depolarization of mitochondrial membrane potential (MMP) of PC3 prostate cancer cell lines was induced by the active extracts of *Marantodes pumilum*. PC3 cell lines were treated with the IC50 of MPc and MPh or Camptothecin (Camp) for 6 h. (**A**) Representative MMP profiles of flow cytometry for active plant extract-treated PC3 cells. (**B**) Quantification of depolarization intensity. The data are means ± SEMs (*n* = 3). * *p <* 0.05 and ** *p <* 0.01, against control, were determined via unpaired *t*-test.

Mitochondrial changes, including variations in mitochondrial membrane potential (Δψm), are the critical events during drug-mediated apoptosis [11]. Figure 6 shows that MPc and MPh increased the percentage of mitochondrial membrane potential depolarization in PC3 cell lines. This could suggest that the active extracts of the plants induce cell death in human prostate cancer cells with the involvement of mitochondrial membrane depolarization.

### 2.6. Inhibition of PC3 Cells Migration

**Figure 7:**
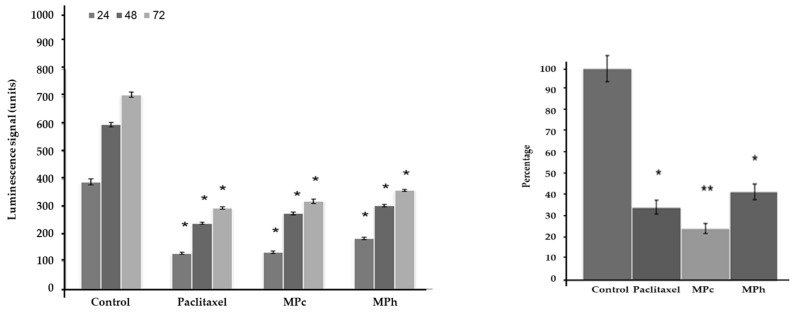
Effect of extracts on PC3 cells migration and invasion. Graphs show 2D horizontal cells migration (ORIS assay, (**left**); cells treated with the MNTC concentration of MPc and MPh for 24, 48, and 72 h); CytoSelect migration assay, (**right**) (cells treated with the MNTC concentration of MPc and MPh for 48 h). The results shown are representatives of three independent experiments. Data are means ± SEMs (*n* = 3). * *p* < 0.05 and ** *p* < 0.01, as determined via unpaired *t*-test.

Figure 7 (left) indicates that MPc and MPh extracts suppressed the migration of PC3 cells in a time-dependent manner. The MPc extract reduced the number of migrated cells by 50% at every time point (Figures S7–S9, Supplementary Materials). To further validate the results of this study, another migration study was conducted using the trans-well migration assay method (Boyden Chamber). Results obtained from this experiment are shown in Figure 7 (right). After 24 h of treatment with MPc, the number of migrated PC3 cells has reduced significantly, by almost 60%.

### 2.7. Inhibition of PC3 Cells Invasion

**Figure 8:**
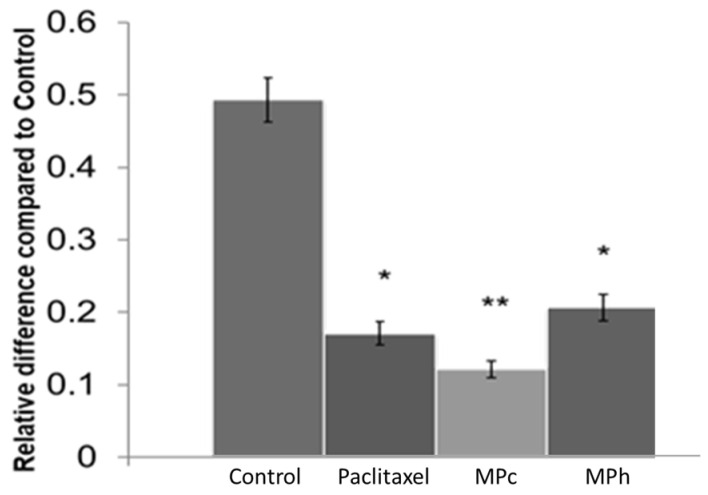
Effect of extracts on PC3 cells using the CytoSelect invasion assay (cells treated with the MNTC concentration of MPc and MPh for 48 h). The results shown are representatives of three independent experiments. Data are means ± SEMs (*n* = 3). * *p* < 0.05 and ** *p* < 0.01, as determined via unpaired *t*-test.

Figure 8 shows the effect of both MPc and MPh on PC3 cell invasion after 48 h of treatment. Both active extracts exhibited significant inhibition of the invasion of the prostate cancer cells after 48 h of treatment. In addition, MPc and MPh extracts reduce the number of invading cells fourfold and twofold, respectively, compared to the control. These results indicate that both MPc and MPh are not only capable of inhibiting or delaying PC3 cell migration but could also prevent cell invasion in a very significant way.

### 2.8. Modulation of Migration and Invasion-Related mRNA Gene Expression

Both MPc and MPh extracts inhibit the mRNA gene expression of both CXCL12 and VEGF-A (Figure 5) in a significant manner (*p <* 0.001). However, neither MPc nor MPh significantly affected the CXCR4 mRNA gene expression. The significant inhibition of the mRNA gene expression of both CXCL12 and VEGF-A by MPc and MPh suggests the potential underlying molecular mechanisms for their in vitro anti-migratory and anti-invasive activities.

### 2.9. Phytochemical Results

#### 2.9.1. Bioguided Isolation of the Active Fraction

The MPc extract was chosen for further fractionation based on the IC50 value. One hundred forty fractions were collected for the MPc, and each fraction was examined via thin-layer chromatography (TLC). Based on the TLC profiles of MPc, the eluates were then pooled into seven major fractions (fraction 1, fraction 2–8, fraction 10–13, fraction 15–17, fraction 21–24, fraction 27–29, fraction 30–33). These fractions were subjected to another cytotoxicity study with PC3 cell lines. Fraction MPc 30–33 showed improved cytotoxicity with the lowest IC50 (4.1 μg/mL) value compared to the other six fractions. Therefore, it was chosen for further microfractionation processing with HPLC. Based on the HPLC chromatograph (Figures S10 and S11, Supplementary Materials), eight microfractions were collected from MPc F30–33, and all of these microfractions were subjected to a cytotoxicity assay on PC3 cells using SRB. The IC50 of each microfraction was calculated, and only microfraction 5 (MF5) was significantly cytotoxic (*p* < 0.05) against PC3 cells with an IC50 value of 4 μg/mL.

#### 2.9.2. Elucidation of the Active Compound

Microfraction 5 was further analyzed with NMR, MS, and FTIR to identify the active compound (data presented in Supplementary Materials).

**Figure 9:**
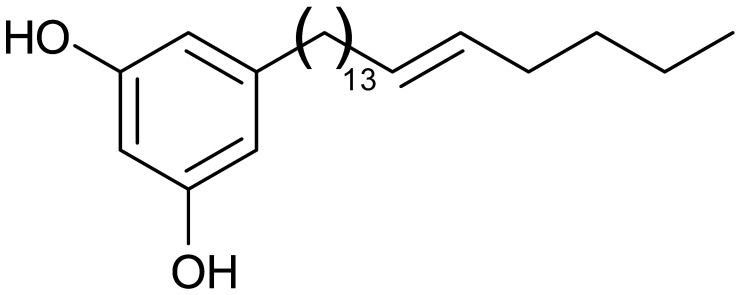
Structure of MP-1.

Compound MP-1, shown in Figure 9, was isolated as a colorless oil. In the IR spectrum, the absorption bands for hydroxyl were observed at 3380 cm^−1^ and alkenyl at 1600 cm^−1^ (Figure S12, Supplementary Materials). The HRESI-MS (Figure S13, Supplementary Materials) of compound MP-1 exhibited a protonated molecular ion [M+H]^+^ peak at *m*/*z* 402.2 (calculated for C_27_H_45_O_2_+H, 401 Da), which corresponds to the molecular formula C_27_H_45_O_2_. The ^1^HNMR and ^13^CNMR spectra (Figures S14–S20, Supplementary Materials) gave signals consistent with 5-henicosene-1-yl-resorcinol.

### 2.10. Cytotoxicity towards Human Dermal Fibroblasts Cells (HDFa)

This experiment investigates the effect of active plant extracts on normal human cells. The IC80 of both MPc and MPh is about 200 μg/mL. For the isolated compound (MP-1), the IC80 is more than 100 μg/mL, considerably lower than its active extract, MPc. However, considering the very low IC50 value of MP-1 (4 μg/mL), the isolated compound is still considered to show selective cytotoxicity, thus indicating that all the active extracts of the plant, as well as the isolated compound, show good selectivity towards prostate cancer cell lines.

### 2.11. Modulation of mRNA Gene Expression by MP-1

#### 2.11.1. Apoptosis-Related Gene Expression

MP-1 significantly inhibited Bcl-2 and ALOX-5 mRNA gene expression by more than twentyfold (*p <* 0.001) and threefold (*p <* 0.05), respectively. MP-1 significantly increased the expression of Bax and Smac/DIABLO mRNA gene expression by 20- and 50-fold, respectively (*p <* 0.001) (data not shown). Therefore, these data suggest that MP-1 induces PC3 prostate cancer cell death via apoptosis, as observed with their active extracts.

#### 2.11.2. Migration-Related Gene Expression

MP-1 significantly downregulated the mRNA gene expression of VEGF-A by 22-fold (*p <* 0.001). In addition, even though MP-1 does not cause any significant changes to the expression of CXCR4, it significantly downregulated CXCL12 expression by more than 20-fold (*p <* 0.001). These findings are similar to the previous results observed with the active extracts of the plants but demonstrate greater efficacy. Therefore, MP-1 also inhibits PC3 prostate cancer cell migration and invasion through a similar mode of action.

## 3. Materials and Methods

### 3.1. Plant Materials Extraction and Fractionation

Certified dried plant materials of *Marantodes pumilum* (Blume) Kuntze (synonyms *Labisia pumila* (Blume) Fern.-Vill.; *Labisia pumila* (Blume) Mez.) were obtained from dedicated farms in Southern Malaysia. All the plant materials were authenticated by Mr. Husnui Hanani Solb at Universiti Putra Malaysia, Malaysia. A voucher (SK 2313/13) was deposited at the Institute of Bioproduct Development, Universiti Teknologi Malaysia (Malaysia). Samples were air-dried and powdered using a laboratory-scale mill and then extracted with methanol for 72 h via a maceration process. The crude extracts were obtained after the evaporation of the methanol to complete dryness under reduced pressure at 40 °C. The crude methanol extract was redissolved in 90% methanol and partitioned into *n*-hexane (MPh extract), chloroform (MPc extract), and water (MPa extract).

All plant extracts were fingerprinted using HPLC-DAD as follows: all samples were dissolved in methanol ≥99.9% for HPLC (Sigma-Aldrich^®^, Gillingham, UK)) and mixed properly using an ultrasonic bath. The concentration of each extract was equivalent to 1 mg/mL. A volume of 5 mL of each sample was filtered through a 0.45μm filter before analysis. The rest were evaporated to dry and stored in a freezer. The filtered samples (10 μL) were injected for HPLC analysis in an Agilent 1200 series (Agilent, Stockport, UK). The stationary phase was Agilent C18 column (250 mm × 4.6 mm id, 5 μm). Samples were eluted with a mobile phase consisted of formic acid solution (A, 0.1 % *v*/*v*) and acetonitrile (B, ≥99.9% for HPLC, (Sigma-Aldrich^®^, Gillingham, UK)) using a linear gradient program (5% B in 0–5 min, 5–15% B in 5–20 min, 15–20% B in 20–35 min, 20–35% B in 35–45 min, 35–100% B in 45–60 min). The flow rate was 1.0 mL/min and the chromatogram was detected at wavelengths of 210 nm, 269 nm, 280 nm, 365 nm. The data was collected and processed with the Agilent Chemstation© Edition software (Agilent, Stockport, UK).

A sample (500 mg) of the crude chloroform extract was suspended in 2 mL of absolute methanol and applied onto a chromatographic column (2.3 × 40 cm) packed with Sephadex LH-20 (Sigma-Aldrich^®^, Gillingham, UK) and equilibrated with absolute methanol. The column was exhaustively washed with absolute methanol at a flow rate of 60 mL/h. A total of 140 6 mL fractions each were collected using a Retriever ^®^ II fraction collector. All fractions were examined via TLC (Silica gel 60 F_254_, Merck KGaG, Taufkirchen, Germany) using Chloroform–Methanol (80:20, *v*/*v*) or Toluene–Ethyl Acetate–Acetic Acid (80:18:2, *v*/*v*/*v*) as mobile phases. The bands were visualized under white light at 254 nm and 366 nm wavelengths before and after derivatization with anisaldehyde using a TLC Visualizer (CAMAG, Muttenz, Switzerland). Eluates were then pooled into major fractions based on their TLC profiles. After the evaporation of methanol, the samples were left to be air-dried, and the residues were weighed.

The bioactive compound was Isolated following multiple microfraction collection using an Agilent 1200 series HPLC-DAD system (Agilent, Stockport, UK) equipped with a C18 column (250 mm × 4.6 mm id, 5 μm) and an Agilent 1200 series fraction collector. The data were collected and processed with the Agilent OpenLab C.D.S. Chemstation Edition software (Agilent, Stockport, UK).

### 3.2. Identification of the Bioactive Compound Using Nuclear Magnetic Resonance (NMR), Mass Spectrometry, and Fourier Transformed Infrared Spectroscopy (FT-IR)

Samples for NMR experiments were prepared in 0.8 mL of Benzene-d_6_. Two-dimensional (2D) NMR spectra were obtained using a Bruker Avance 500 MHz NMR spectrometer (Bruker, Coventry, UK) equipped with broadband and a triple resonance (^1^H, ^13^C and ^15^N) inverse probe. 2D experiments carried out included COSY (Correlation Spectroscopy), HMQC (Heteronuclear Multiple-Bond Correlation), HMBC (Heteronuclear Multiple-Bond Correlation), and NOESY (Nuclear Overhauser Effect Spectroscopy).

The molecular weight of the isolated compound was determined via electrospray ionization mass spectrometry (ESI-MS), using both positive and negative modes on a LCQ Duo Ion-Trap mass spectrometer (Thermo Fisher, Altrincham, UK). The sample was run in 50%_aq_ methanol as a mobile phase under the following conditions: sheath gas flow rate (20–100 units), auxiliary gas flow rate (0–60 units), ion spray voltage (1–8 K.V), spray current (0.34 µA), capillary temperature (100–220 °C), capillary voltage (38–100 V), and tube lens offset (0–40 V).

The FT-IR spectrum was recorded on a Spectrum 100 FT-IR Spectrometer as a thin film (Perkin Elmer, Beaconsfield, UK).

### 3.3. Cell Culture

The PC-3 cell line (ATCC Number: CRL-1435™) was kindly provided by Dr. Cyrill Bussy (Centre for Drug Delivery Research, UCL School of Pharmacy, UK), the LNCaP clone FGC cell line (ATCC Number: CRL-1740™) was purchased from Sigma Aldrich (Gillingham, UK), and was obtained from the American Type Culture Collection (Teddington, UK). Human Dermal Fibroblasts, adult (HDFa) catalogue number C-013-5C was kindly provided by Dr. George Pasparakis (Department of Pharmaceutics, UCL School of Pharmacy).

Both PC-3 and LNCaP cell lines were grown in a Nunclon™ cell culture flask (Nunc, UK), with a surface area of 75 cm^2^, and maintained in RPMI-1640 (Lonza, Slough, UK)-containing L-glutamine. The media were supplemented with 10% of heat-inactivated FBS (Gibco, UK) and 1% penicillin–streptomycin antibiotics containing 10,000 units/mL of penicillin and 10,000 μg/mL of streptomycin (Gibco, Altrincham, UK). The HDFa cell line was grown in similar flasks using DMEM supplemented with L-glutamine, high glucose, 10% of heat-inactivated FBS, 1% 100× NEAA, and 0.1% of both 10 mg/mL gentamicin solution 1000× and 250 ug/mL amphotericin B solution 1000× (Gibco, UK). All cells were maintained at 37 °C in a humidified atmosphere of 5% CO_2_. The prepared media were used to grow and seed the cells in a Nunclon™ 96-well plate (Nunc, UK) for cell-based assays, plant extracts, and fractions dilution.

### 3.4. Cell Viability Assays

The Sulforhodamine B (SRB) assay and MTT assays were performed as previously described [12,13]. All reagents were from Sigma-Aldrich (UK).

The SRB assay determined the antiproliferative activity of the extracts. This assay was performed according to a previously described method [14]. A375 cells were seeded at a density of 10,000 cells/well in a 96-well plate (Thermo Fisher, Altrincham, UK) and left overnight to attach at 37 °C. Afterward, cells were treated with a serial dilution of the plant extracts (200, 100, 50, 25, 12.5, 6.25 µg/mL) at several time points. Upon the completion of the incubation period, the cells were fixed with a trichloroacetic acid solution for one hour at 4 °C. After washing with water, cell proteins were stained with SRB solution and left at room temperature for one hour. The plate was washed four times with 1% acetic acid and flicked to remove unbound dye. Then, Tris-based buffer solution was added to each well, and the absorbance was measured at 510 nm. Cell growth was calculated using the following equation:

$$\%\mathrm{Cell}\;\mathrm{growth}=\frac{\mathrm{Absorbance}\;\left(\mathrm{sample}\right)-\mathrm{Absorbance}\;\left(\mathrm{blank}\right)}{\mathrm{Absorbance}\;\left(\mathrm{vehicle}\;\mathrm{control}\right)-\mathrm{Absorbance}\;\left(\mathrm{blank}\right)}\times 100$$

In the MTT assay, 10 µL of the MTT solution (5 mg/mL dissolved in PBS) was added to all wells after incubation and then further incubated for 4 h. After 4 h in a humidified atmosphere at 37 °C, both the cell media and the MTT solution were removed from the wells, and 200 µL of DMSO was added in each well to allow the dissolution of the purple MTT-formazan crystals. The absorbance (optical density, OD) was measured at 570 nm and a reference wavelength of 630 nm with a microplate reader (Tecan Infinite^®^ M200). The relative difference to control was determined using the following equation:

$$\mathrm{Relative}\;\mathrm{difference}\;\mathrm{to}\;\mathrm{control}=\frac{\mathrm{OD}\;\left(\mathrm{sample}\right)\;-\;\mathrm{OD}\;\left(\mathrm{Blank}\right)}{\mathrm{OD}\;\left(\mathrm{Control}\right)-\mathrm{OD}\left(\mathrm{Blank}\right)}$$

### 3.5. Cell Microscopy

PC3 and LNCaP cells were seeded in 12-well plates and left to attach and proliferate for 48 h of incubation time. Then, the plant extracts were added, and the morphology and the population of the cells were monitored using an EVOS^®^ FL Imaging System at 24, 48, and 72 h either with or without 4′,6-Diamidino-2-phenylindole (DAPI) for nucleic acid staining (Sigma-Aldrich, UK).

### 3.6. Cell Cycle Distribution Study

PC3 and LNCaP cells were seeded in 6-well plates and incubated for 48 h. Plant extracts and paclitaxel (positive control) were added after 48 h, and the cells were further incubated for 48 h at 37 °C in a 5% CO_2_ atmosphere. The cells were then washed and trypsinized with 0.25% Trypsin-EDTA solution for cell detachment. Cell pellets were resuspended in 1 mL PBS and washed twice by adding 10 mL PBS. Next, the cells were centrifuged at 300× *g* for 10 min at 4 °C. Once the supernatant was removed, 1 mL of ice-cold 70% ethanol was slowly added drop by drop to the cell pellet. The cells were allowed to fix in ethanol for 18 h. After 18 h, the cells were centrifuged at 500× *g* for 10 min at 4 °C. The supernatant was removed, and 1 mL of a staining solution containing 1 mg/mL propidium iodide and 100 Kunitz units/mL of RNase A was added to the cells and incubated for 30 min before being analyzed using a MACSQuant^®^ Analyzer 10 flow cytometer.

### 3.7. Apoptosis Detection Assays

#### 3.7.1. Caspases 3/7 Activity

In this study, the apoptosis induced by plant extracts was determined by measuring the activity of caspases 3 and 7 using an apoptosis detection kit (Promega, G8091). The Caspase-Glo^®^ 3/7 assay was performed according to the manufacturer’s protocol. First, PC-3 and LNCaP cells were seeded in 96-well plates at 2.5 × 10^3^. The cells were left for 24 h to allow cell attachment. After 24 h, the cells were treated with extracts, fractions, vehicle control (DMSO), paclitaxel, and a blank (cell-free medium) for 48 h. Next, 100 μL of Caspase-Glo^®^ 3/7 reagent was added to each well after 48 h of incubation and mixed gently for 30 s. Then, the plate was incubated for 1 h at room temperature. After the completion of the incubation period, the luminescence of each plant extract was measured using an Infinite^®^ M200 plate-reading luminometer (Tecan, Männedorf, Switzerland). The assay was performed independently in triplicate, and the results were calculated using the following equation:

R.L.U. = Luminescence (samples) − Luminescence (blank)

#### 3.7.2. Annexin V-FITC and Propidium Iodide Staining

PC3 and LNCaP cells were seeded in 12-well plates and incubated for 48 h. After incubation, plant extracts and paclitaxel (positive control) were added and further incubated for 6 h at 37 °C in a 5% CO_2_ atmosphere. The cells were washed with PBS and detached with 0.25% Trypsin-EDTA solution. The cells were resuspended in 100 μL of 1× binding buffer, and 10 μL of annexin V-FITC was added. The mixture was mixed carefully and incubated for 15 min in the dark at room temperature. After 15 min, the cells were washed with 1 mL of 1× binding buffer and centrifuged at 300× *g* for 10 min. Subsequently, the cells were resuspended in 500 μL of 1X binding buffer and propidium iodide (PI) solution was added before flow cytometry (MACSQuant^®^ Analyzer 10) analysis. The annexin-V FITC kit was from Miltenyi Biotec (Frimley Green, UK).

#### 3.7.3. Determination of Mitochondrial Membrane Potential

The change in mitochondrial transmembrane potential (Δψ_m_) induced by the active extracts of the plants in the prostate cancer cell line (PC3) was determined by using a Mito Probe™ JC-1 Assay Kit (ThermoFisher Scientific, UK) [15]. Briefly, 1 × 10^6^ cells/mL of PC3 were seeded into 6-well plates and incubated for 48 h. After incubation, plant extracts and Carbonyl Cyanide Chlorophenylhydrazone (CCCP) as a positive control were added and further incubated for 6 h at 37 °C in a 5% CO2 atmosphere. The treated cells were then labeled with 2 μM of JC-1 for 15 min at 37 °C and washed with warm phosphate-buffered saline (PBS). The cells were analyzed on a MACSQuant^®^ Analyzer 10 flow cytometer with 488-, 530-, and 585-nm pass emission filters. CCCP-treated cells (10 μM) were taken as positive controls.

#### 3.7.4. Terminal Deoxynucleotidyl Transferase-Mediated Biotin dUTP Nick End-Labeling Assay

We used the Dead-End apoptosis detection kit (dUTP Nick End Labeling, TUNEL assay) from Promega (Madison, USA) to detect apoptotic cells via in situ end-labeling of the 3′OH end of the DNA fragments generated by apoptosis-associated endonucleases. Briefly, the cells were grown in a LabTek II chamber slide and treated with plant extracts for 72 h. First, the cells were washed in phosphate-buffered saline and fixed by immersing the slides in 4% paraformaldehyde for 25 min. All the steps were performed at room temperature unless otherwise specified. Next, they were washed twice by immersing them in fresh phosphate-buffered saline for 5 min. Next, cells were permeabilized with 0.1% Triton X-100 solution in phosphate-buffered saline for 5 min, washed twice in phosphate-buffered saline, and then covered with 100 μL of equilibration buffer and kept for 5–10 min. Finally, the equilibrated areas were blotted around with tissue paper, and 50 μL of terminal deoxynucleotidyl transferase (TdT) reaction mix was added to the slides’ sections and then incubated at 37 °C for 60 min inside a humidified chamber for the end-labeling reaction to occur. Immersing the slides in a 2× sodium chloride–sodium citrate buffer for 15 min terminated the reactions. Next, the slides were washed thrice by immersing them in fresh phosphate-buffered saline for 5 min to remove unincorporated biotinylated nucleotides. The slides were then immersed in a freshly prepared 1 μg/mL propidium iodide solution for 15 min at room temperature in the dark. The washing procedure was repeated after 15 min. Then, the slides were immediately analyzed under a fluorescence microscope (EVOS^®^F.L. Imaging System, Thermo Fisher, Altrincham, UK) using a standard fluorescein filter set to view the green fluorescein at 520 ± 20 nm) and propidium’s red fluorescence iodide at 620 nm.

### 3.8. In Vitro Cell Motility Assays

#### 3.8.1. Cell Migration Assays

The in vitro cell horizontal migration was determined using the Oris (96-well cell migration assay kit (Platypus Technologies, Fitchburg, WI, USA). 5 × 10^4^ PC3 cells were seeded in each well and left for 24 h. The stoppers that were used to create the migration zone were removed after 24 h, and the cells were washed with PBS to remove any unattached cells. Then, 100 μL of new media, with or without the plant extracts, were added to each well. The cells were allowed to migrate into the migration zone for 72 h. Cells were stained with CellTracker™ Green (Life Technologies, Carlsbad, CA, USA). The seeded plate was incubated in a humidified chamber for 72 h, and at various time points (24 h, 48 h, 72 h), the fluorescent signals in the detection zone were measured using a microplate reader (Synergy™ HT, BioTek) with 492-nm excitation and 517-nm emission filters.

The transversal cell migration study (Boyden chamber) was also performed using the CytoSelect Cell Migration Assay kit (Cell Biolab, San Diego, CA, USA) [16]. This kit contains polycarbonate membrane inserts (8-μm pore size) in a 24-well plate. Under sterile conditions, the 24-well migration plate was allowed to warm up to room temperature for 10 min. A cell suspension containing 1.0 × 10^6^ cells/mL in serum-free media was prepared. Fresh media (control) and media with respected plant extracts were added directly to individual trans-well inserts with the cell suspension. Overnight serum starvation was performed before running the assay. Then, 500 μL of media containing 10% fetal bovine serum was added to the lower well of the migration plate, and 300 μL of the cell suspension solution was added to the inside of each insert and then incubated for 24 h in a cell culture incubator. After 24 h of incubation, the media were carefully aspirated from the inside of the trans-well insert. The interior part of the inserts was washed with a wet cotton swab to remove non-migratory cells. The inserts were transferred to a clean well containing 400 μL of Cell Stain Solution (crystal violet dye) and incubated for 10 min at room temperature. Then, the inserts were gently washed in a beaker of water and left to air dry. Each insert was transferred into wells containing 200 μL of Extraction Solution (10% Acetic Acid) and incubated for 10 min at room temperature on an orbital shaker. Then, 100 μL of each sample was transferred to a 96-well plate and measured at 560 nm using an Infinite^®^ M200 microtiter plate reader (Tecan, Männedorf, Switzerland).

#### 3.8.2. Cell Invasion Assay

The cell invasion was measured using the same Cytoselect 24-well cell invasion assay kit (Catalogue number CBA-100-C, Cell Biolabs, Inc., San Diego, CA, USA) with the variation of coating the upper surface of the insert membrane with a protein matrix isolated from Engelbreth–Holm–Swarm tumor cells. Then, the basement membranes of Boyden chambers were rehydrated with 300 μL serum-free media, and 1 × 10^6^ cells were then seeded into the upper area of the chamber in serum-free media (control) with or without the plant extracts. Overnight serum starvation had been performed prior to running the assay. Then, 500 μL of media containing 10% FBS was added to the lower well of the migration plate. After incubation for 48 h, the non-invading cells on the upper surface of the inserts were removed with a cotton swab and invading cells on the lower surface were stained with crystal violet Cell Stain Solution and incubated for 10 min at room temperature. Then, the inserts were gently washed in a beaker of water and left to air dry. Each inserts was transferred into wells containing 200 μL of Extraction Solution (10% acetic acid) and incubated for 10 min at room temperature on an orbital shaker. Then, 100 μL of each sample was transferred to a 96-well plate and measured at 560 nm by using an Infinite^®^ M200microtiter plate reader (Tecan).

### 3.9. Real-Time RT-qPCR Analysis

#### 3.9.1. mRNA Extraction and cDNA Synthesis

After exposing PC3 cells (5 × 10^5^ cells/well) to plant extracts, fractions, and DMSO 1% for 96 h, the total RNA was extracted using RNeasy^®^ Plus Mini (Qiagen, Manchester, UK) according to the manufacturer’s protocol. Samples were treated with a gDNA eliminator spin column to avoid genomic DNA contamination. The quantity and quality of RNA were determined by differential readings at 260- and 280-nm wavelengths using Nanodrop 2000 (Thermo Scientific). The integrity of total RNA from PC3 cells was assessed via visual inspection of the two rRNAs for 28 and 18 s on agarose gels. cDNA was synthesized from 1 µg of total RNA using Omniscript^®^ Reverse Transcription kit according to the manufacturer’s instruction in a final volume of 20 µL.

#### 3.9.2. RT-qPCR Conditions and Analysis

The sequence for the primers used in this study is listed in Table 2. All primers used in this study were designed and obtained from Primerdesign Ltd. (Eastleigh, UK). The RT-qPCR was carried out in 96-well plates using a PikoReal™ Real-Time PCR detection System (Thermo Fisher, Altrincham, UK). Each well contained a final reaction volume of 20 µL (10 µL of PrecisionFAST™ MasterMix with SYBR Green, 5 µL of cDNA template diluted appropriately, 1 µL of resuspended primer mix at a final concentration of 300 nM, and 4 µL of RNase/DNase free distilled water). The PCR reaction was performed using the following conditions: initial denaturation at 95 °C for 2 min, then 40 cycles of denaturation at 95 °C for 15 s, the corresponding annealing temperature of each gene as listed in Table 1 for 30 s, and extension at 72 °C for 30 s. At the end of the run, heating the amplicon from 60 to 95 °C to confirm the specificity of the amplification for each primer pair generated a melting curve. All RT-qPCRs were run in quadruplicates. Standard curves were produced to check the PCR efficiency using a fivefold dilution series of cDNA. The efficiency (E) of primer pairs was obtained from the slope of the calibration curve generated. The relative expression was calculated based on ‘delta-delta Ct’ (∆∆Ct) values. The target genes were normalized by using GAPDH as a reference gene.

### 3.10. Statistical Analysis

Data collected and reported in this study are expressed as means ± standard deviations. Statistical data analysis was conducted using GraphPad Instat version 3 (GraphPad Software Inc, La Jolla, CA, USA). All experiments were conducted three times independently in triplicate. The Inhibitory concentration 50 (IC50) values were taken from the minimal experimental concentration showing 50% cell death.

## 4. Discussion

Two different cell lines have been used in this study (PC3 and LNCaP), as each cell line represents a different stage of prostate cancer. The LNCaP cancer cell line represents androgen-dependent prostate cancer, whereas PC3 cell lines represent androgen-independent prostate cancer [17]. According to recent statistics, many patients die as therapy eventually fails when the disease progresses to the androgen-independent stage [18]. In this study, our focus is to investigate the effect of the active plant extracts on PC3 cells, a type of androgen-independent prostate cancer cell. Our data show that all active plant extracts and their isolated compound are cytotoxic against both types of prostate cancer cell lines. Al-Mekhlafi and coworkers already described the antiproliferative properties of *Marantodes pumilum* extracts against certain cancer cell lines, such as colon, breast, and prostate cancer cell lines, but did not study their possible mechanisms of action [8]. We present the first detailed account of such activity: that MPc and MPh extracts and the isolated MP-1 compound induce PC3 cell death via apoptosis through the intrinsic pathway. This is evidenced by the significant activation of caspases 3 and 7. They also affect the mRNA gene expression of Bax, Bcl-2, and Smac/DIABLO. These genes are responsible for the production of their respective proteins, which play a critical role in the intrinsic pathway of apoptosis [19]. Further research is warranted at the protein level to confirm the involvement of these key anticancer targets.

Only MPc and MP-1 induced PC3 cell death via apoptosis independently from the common apoptotic pathways by significantly inhibiting the mRNA gene expression of ALOX-5, which is responsible for the production of the ALOX-5 enzyme. According to Ghosh and Myers [10], the inhibition of ALOX-5 would block the production of its metabolites (leukotrienes), thus triggering apoptosis in prostate cancer cell lines. Furthermore, in PC3 cells treated with the active extracts and MP-1, the cell population in the G0/G1 phase was reduced, whereas the cell population in the G2/M phase increased significantly compared to the untreated PC3 cells. These data implied that all active plant extracts and MP-1 induced cell cycle arrest at the G2/M phase. Cell cycle arrest enables DNA repair to occur, thus preventing replication of the damaged templates, and cell cycle arrest, specifically at the G2M phase, prevents the cells from entering the mitosis (M) phase with damaged genomic DNA [20].

All active plant extracts and the isolated compound MP-1 significantly inhibit the migration and invasion of the PC3 cancer cell line. The CXCL12 and chemokine receptors, CXCR4 and CXCR7, are the key factors that link cancer cells and their microenvironment, thus playing a crucial role in tumor initiation and progression [21], including the migration of prostate cancer cell lines such as PC3 and LNCaP [22]. In our study, the plant extracts MPc, MPh, and the isolate compound MP-1 were able to downregulate the expression of CXCL12 mRNA gene expression significantly, which might lead to the fall in the production of CXCL12 chemokines, thus inhibiting both PC3 cell migration and inhibition. However, none of them were able to have any significant effect on the expression of CXCR4. In contrast, they all significantly inhibited the mRNA gene expression of VEGF-A. Vascular endothelial growth factor (VEGF) is a critical regulator of endothelial cell migration by increasing endothelial cell permeability, stimulating proliferation, and promoting migration of phosphatidylinositol-3-kinase and the small GTPase Rac-1. In addition to these, VEGF also plays a significant role in angiogenesis [23]. Despite the cytotoxic effect of all the active plant extracts and their isolated compound on prostate cancer cell lines, they showed specific selectivity when tested with normal human dermal fibroblast cells (HDFa).

MP-1 is spectroscopically consistent with 5-henicosene-1-yl-resorcinol. Al-Mekhlafi and coworkers previously isolated from this plant several alkylresorcinols with cytotoxic activity against PC3 and other cancer cells [8]. One of them, labisiaquinone A, is virtually a dimer of 5-henicosene-1-yl-resorcinol, possibly indicating a close relationship within the secondary metabolism of the plant. Alkylresorcinols are present in higher plants, bacteria, fungi, algae, and mosses and show anti-parasitic, anti-fungal, anti-microbial, antioxidant, and cytotoxic effects [24,25,26]. In particular, the cytotoxic activity of 5-alkylresorcinol compounds on cancer cell lines has been reported in several studies previously, such as in the cancer cell lines of the breast [27], colon [8], prostate [8,27], liver [28], and human squamous carcinoma [29]. Using leukemia KB cells, it was evidenced that the cytotoxic activity increased with the number of carbons on the side chain (from 5 to 13) and that hydroxylation at C1 and C3 was a structural requirement for cytotoxicity [30]. In this study, we have added 5-henicosene-1-yl-resorcinol to the list of cytotoxic alkylresorcinols and provided the first detailed report on some of the cellular and molecular mechanisms of this cytotoxic and antimigratory class of compounds.

## 5. Conclusions

In conclusion, the chloroform and hexane extracts of *Marantodes pumilum* and the isolated 5-henicosene-1-yl-resorcinol were able to overcome three hallmarks of cancer in PC3 cells: (1) apoptosis by activating the intrinsic pathway, (2) inhibition of both migration and invasion, and (3) inhibiting angiogenesis.

## Figures and Tables

**Table 1:** Cytotoxic activity (IC50 values in µg/mL) of the extracts against the selected prostate cancer cell lines. MTT measures mitochondrial viability, and SRB measures cell proliferation.

Treatment	Code	MTT Assay		SRB Assay		
		DU145	LNCaP	PC3	DU145	LNCaP
n-Hexane	MPh	24.57	24.86	20.13	25.14	27.53
Chloroform	MPc	13.14	13.76	14.26	14.29	13.76
Aqueous	MPa	150	136.2	>200	>200	>200

**Table 2:** Details of the primers used in this study.

Gene	Primer Sequences	Annealing (°C)
GAPDH	Sens: 5′-ATGCTGGCGCTGAGTACGTC-3′ Anti-sense: 5′-GGGCAGAGAGATGATGACCCTT-3′	55
B.A.X.	Sense: 5′-ATGGAGCTGCAGAGGATGAT-3′ Anti-sense: 5′-CAGTTGAAGTTGCCGTCAGA-3′	56.5
BCL-2	Sense: 5′-GAGGTCACGGGGGCTAATT-3′ Anti-sense: 5′-GAGGCTGGGCACATTTACTG-3′	56.8
ALOX5	Sense: 5′-AAGCGATGGAGAACCTGTTCA-3′ Anti-sense: 5′-GTCTTCCTGCCAGTGATTCATG-3′	56.8
VEGFA	Sense: 5′-TGCTCTACTTCCCCAAATCACT-3′ Anti-sense: 5′-CTCTCTGACCCCGTCTCTCT-3′	57.6
Smac DIABLO	Sense: 5′-GCACAGAAATCAGAGCCTCATT-3′ Anti-sense: 5′-TTCAATCAACGCATATGTGGTCT-3′	56.4
CXCR4	Sense: 5′-CCAAAGAAGGATATAATGAAGTCACT-3′ Anti-sense: 5′-GGGCTAAGGGCACAAGAGA-3′	56.4
CXCL12	Sense: 5′-CTCCTCTTTCAACCTCAGTGATT-3′ Anti-sense:5′-GAGAAGCAGAAGCAAGATTAAGC-3′	56.8

## Data Availability

Not applicable.

## Supplementary Materials

The following supporting information can be downloaded at: https://www.mdpi.com/article/10.3390/plants12071576/s1, HPLC fingerprints of *Marantodes pumilum* extracts (Figures S1–S3); Cell morphology (Figures S4–S6); Microscopy images of 2D migration ORIEL assay (Figures S7–S9); HPLC-DAD chromatograms of the microfractions (Figures S10 and S11); Spectroscopic analysis of MP-1 (Figure S12), HR-MS spectra (Figure S13), 1D and 2D NMR spectra (Figures S14–S20) of isolate MP-1.
