# Supplementary material for: In Vitro Pro-Apoptotic and Anti-Migratory Effects of Marantodes pumilum (syn. Labisia pumila) Extracts on Human Prostate Cancer Cell Lines: Bioguided Isolation of 5-Henicosene-1-yl-resorcinol
Source: Plants (Basel). 2023 Apr 6;12(7):1576. doi: 10.3390/plants12071576 (PMC10097395; doi:10.3390/plants12071576)
Supplement: Supplementary file 1 [file plants-12-01576-s001.zip › plants-2099950-supplementary.pdf]

plants

MDPI

*Article*

# In Vitro Pro-Apoptotic and Anti-Migratory Effects of *Marantodes pumilum* (syn. *Labisia pumila*) Extracts on Human Prostate Cancer Cell Lines: Bioguided Isolation of 5-Henicosene-1-yl-resorcinol

**Mohd Mukrish Mohd Hanafi [1], Harisun Yaakob [2], Simon Gibbons [1,3,4] and Jose M. Prieto [1,4,*]**

[1] Department of Pharmaceutical and Biological Chemistry, U.C.L. School of Pharmacy, London WC1N 1AX, UK; mohd.hanafi.12@ucl.ac.uk (M.M.M.H.); s.gibbons@ljmu.ac.uk (S.G.)
[2] Institute of Bioproduct Development (IBD), Universiti Teknologi Malaysia, Johor Bahru 81310, Malaysia; harisun@utm.my
[3] School of Pharmacy, University of East Anglia, Norwich NR4 7TJ, UK
[4] School of Pharmacy and Biomolecular Sciences, Liverpool John Moores University, Liverpool L3 3AF, UK

* Correspondence: j.m.prietogarcia@ljmu.ac.uk

## 1. HPLC Fingerprint analysis of *Marantodes pumilum* extracts.

HPLC-DAD chromatograms were obtained on an Agilent 1200 series HPLC system (Agilent, USA). Fingerprint analysis of plant extracts was conducted as follows: All samples were dissolved in methanol (≥99.9% for HPLC, Sigma-Aldrich) and mixed properly using an ultrasonic bath. The concentration of each extract was equivalent to 1 mg/mL. A volume of 5 mL of each sample was filtered through a 0.45μm filter before analysis. The rest were evaporated to dry and stored in a freezer. The filtered samples (10 μL) were injected for HPLC analysis. The stationary phase was Agilent C18 column (250mm x 4.6mm id, 5μm). Samples were eluted with a mobile phase consisted of formic acid solution (A, 0.1 % v/v) and acetonitrile (B, ≥99.9% for HPLC, Fisher Scientific) using a linear gradient program (5% B in 0–5 min, 5–15% B in 5–20 min, 15–20% B in 20–35 min, 20–35% B in 35–45 min, 35–100% B in 45–60 min). The flow rate was 1.0 mL/min and the chromatogram was detected at wavelengths of 210nm, 269 nm, 280nm, 365 nm. The data was collected and processed with the Agilent Chemstation© Edition software (Agilent).

Figures S1-S3 show the distribution of phytochemical constituents in *Marantodes pumilum* plant extracts. In MP n-hexane extract, even though several peaks were seen between tR =20 to tR =30, most peaks were detected after tR =30. Whereas peak distribution for MP chloroform extract could be detected in two separate regions - the first is between tR =10 to tR =30; and the second region is after tR =40. In the aqueous extract of MP, peaks were only detected in the first 20 minutes of the retention time.

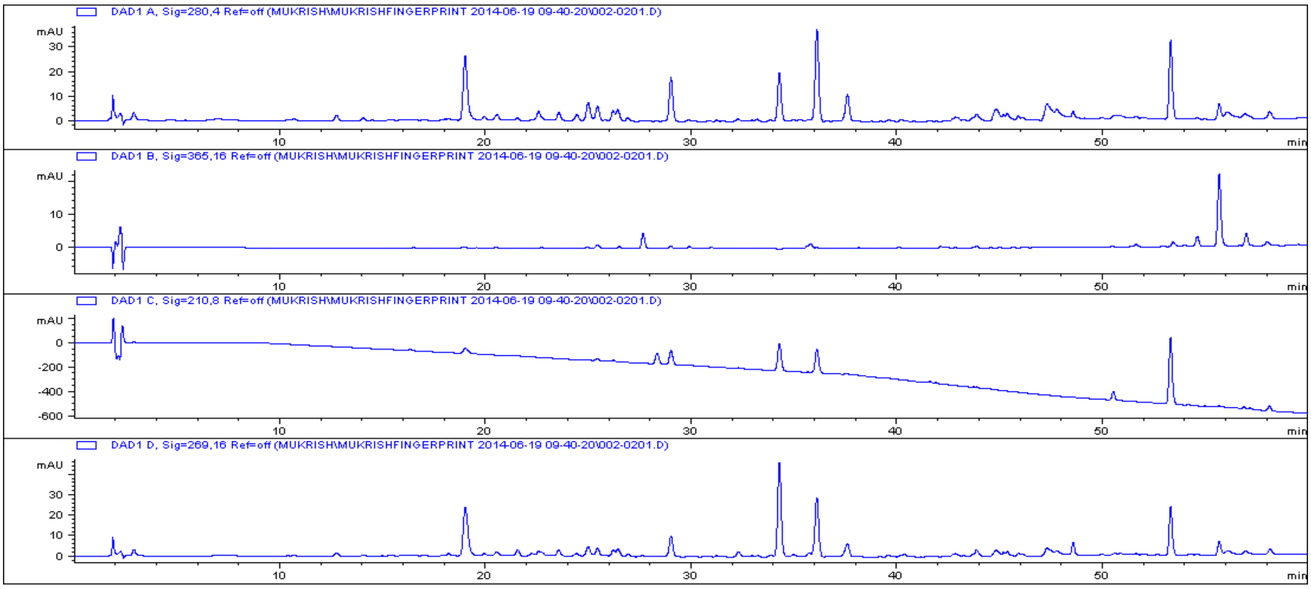


**Figure S1**. HPLC fingerprint of *Marantodes pumilum* hexane extract

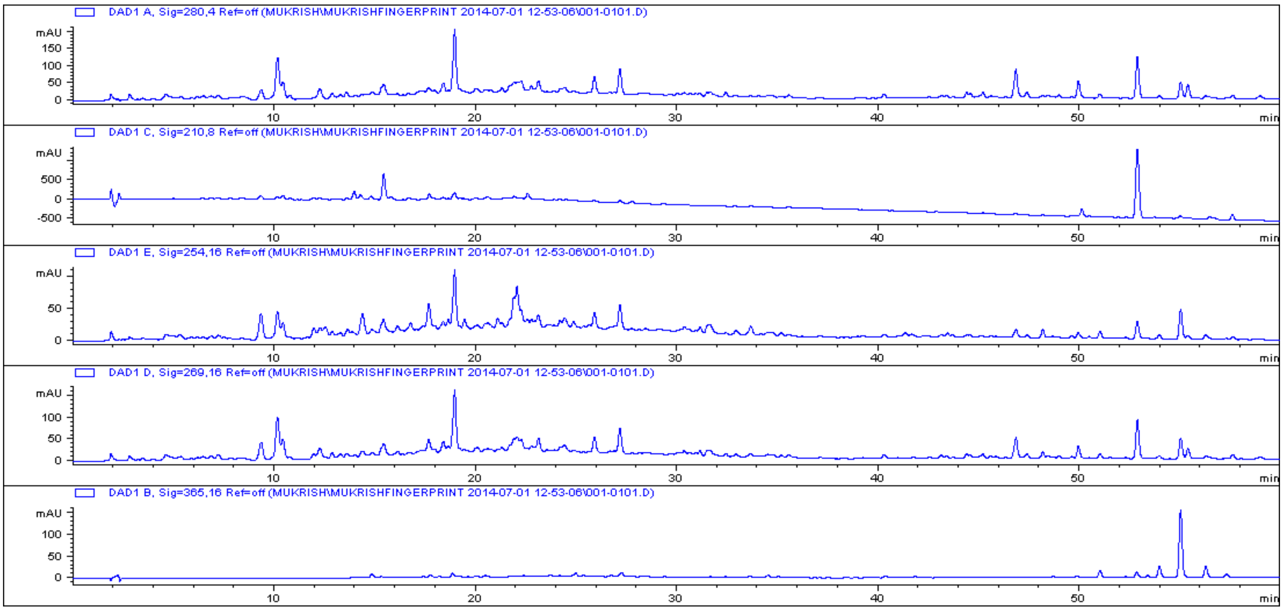


**Figure S2**. HPLC fingerprint of *Marantodes pumilum* chloroform extract

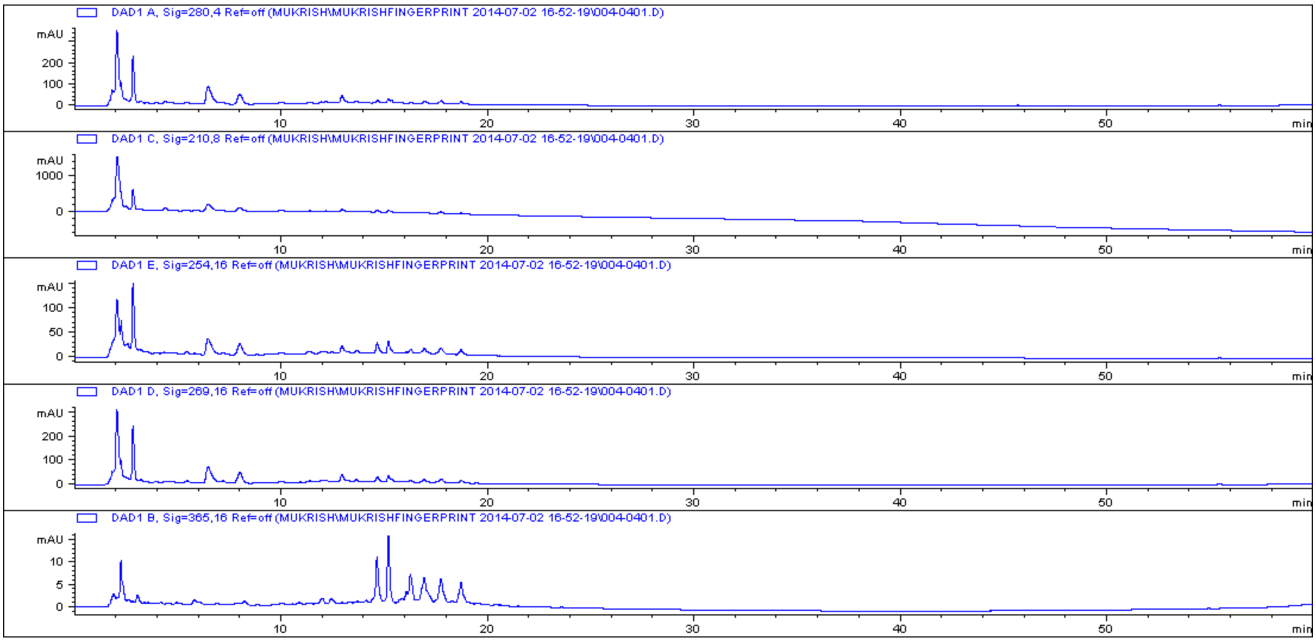


**Figure S3.** HPLC fingerprint of *Marantodes pumilum* aqueous extra

## 2. Cell morphology

In this study, the morphological changes of the prostate cancer cell lines (LNCaP and PC3) untreated and treated with the active extracts of *Marantodes pumilum* were observed using an EVOS®F.L. Imaging System at 24, 48, and 72 hours. Figures S4-S6 show the characteristics of apoptosis, such as cells detachment from the substratum, cell shrinkage, nuclear condensation, membrane blebbing, and the formation of apoptotic bodies, were detected in the treated cells. In addition, the reduction in the cell population was evident when comparing the untreated and the treated cells.

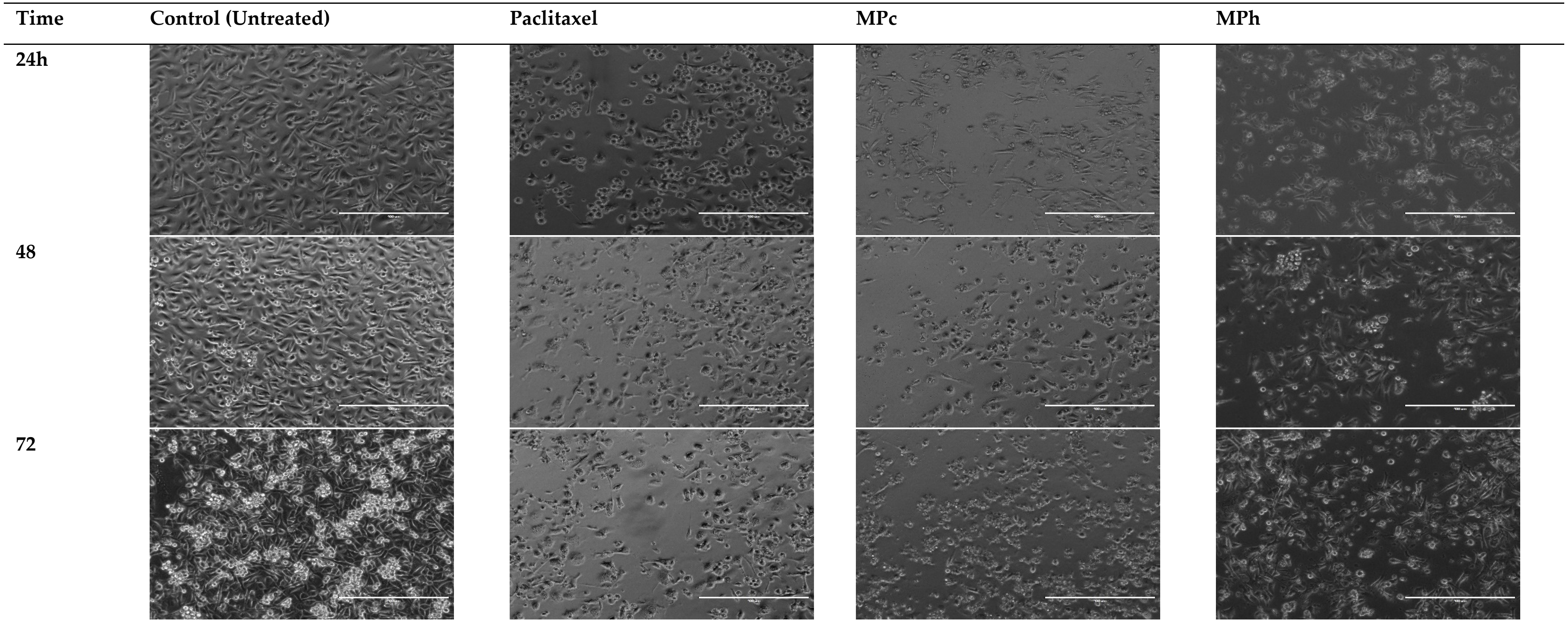


**Figure S4.** Morphological changes of PC3 cells treated with the IC50 of the plant extracts and Paclitaxel (positive control) for 24, 48, and 72 hours viewed under the EVOS® FL Imaging System (100x magnification). Reduction in cell population was noted after 24, 48, and 72 hours of treatment compared to the control (Untreated cells). Images are representative of 3 independent experiments.

| Time | Control (Untreated) | Paclitaxel | MPc | MPh |
| --- | --- | --- | --- | --- |
| 24 | | | | |
| 48 | | | | |
| 72 | | | | |

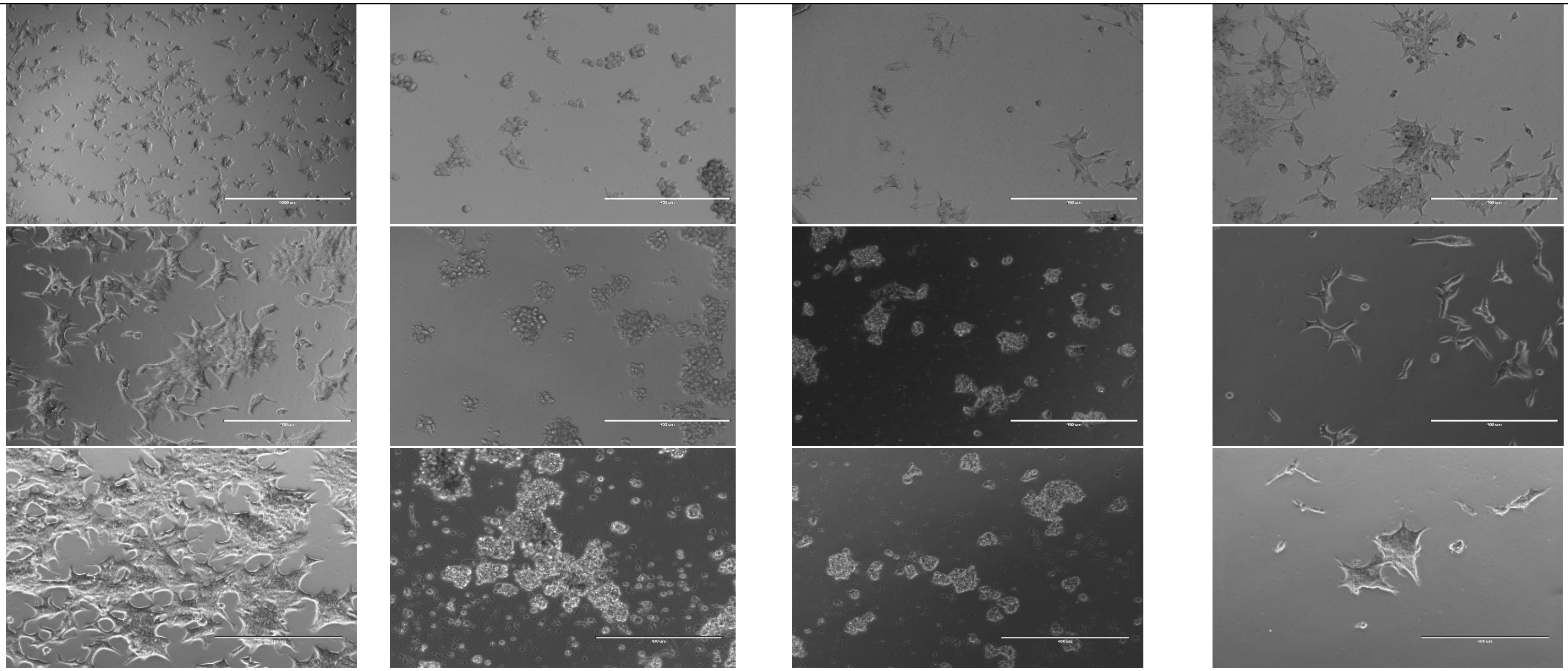

**Figure S5.** Morphological changes of LNCaP cells treated with the IC50 of the plant extracts and Paclitaxel (positive control) for 24, 48, and 72 hours viewed under the EVOS® FL Imaging System (100x magnification). Reduction in cell population was noted after 24, 48, and 72 hours of treatment compared to the control (Untreated cells). Images are representative of 3 independent experiments.

<table>
<tr><th></th><th>Control<br>(Untreated cells)</th><th>Paclitaxel<br>(Reference drug)</th><th>M. Pumilum Chloroform extract<br>(MPC)</th><th>M. Pumilum Chloroform extract<br>(MPC)</th></tr>
<tr><td>PC3</td><td>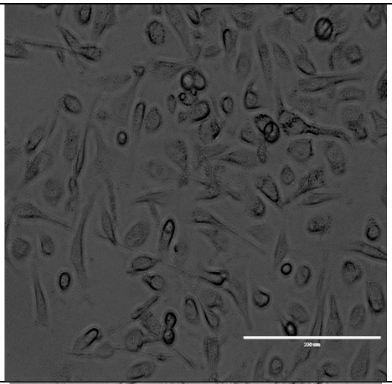</td><td>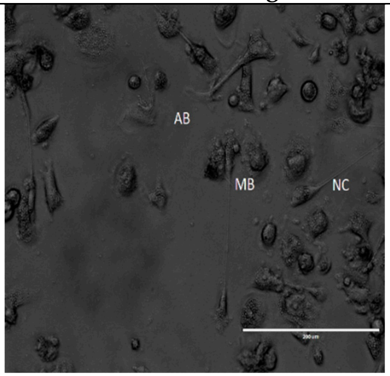
</td><td>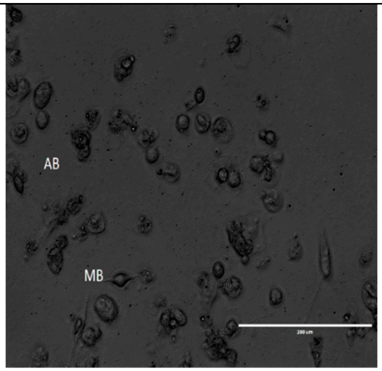
</td><td>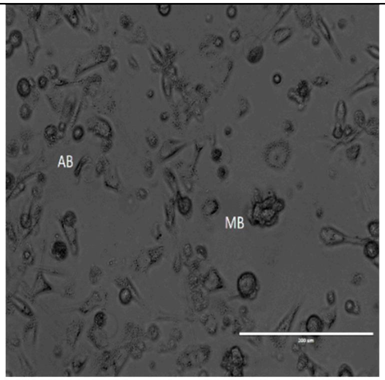
</td></tr>
<tr><td>LNCaP</td><td>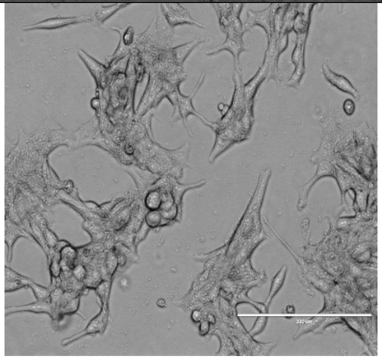</td><td>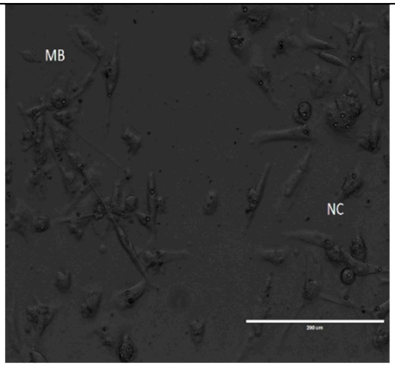
</td><td>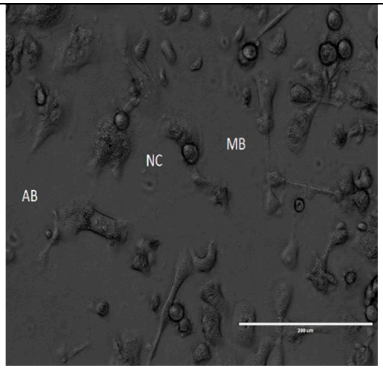
</td><td>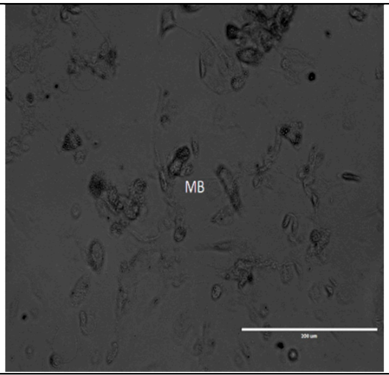
</td></tr>
</table>

**Figure S6.** PC3 (upper row) and LNCaP cells (lower row) viewed under the EVOS® FL Imaging System (200x magnification) after 72 hours of treatment with $IC_{50}$ concentrations of Paclitaxel, *Marantodes pumilum* (Chloroform), and *Marantodes pumilum* (n-Hexane) extracts. The cells showed characteristics of apoptosis, such as the formation of apoptotic bodies (AB), membrane blebbing (MB), and nuclear compaction (NC). Images are representative of 3 independent experiments.

## 3. Microscopy images of 2D migration ORIEL assay

| Sample/Hour | 24 | 48 | 72 |
|---|---|---|---|
| **Control** | 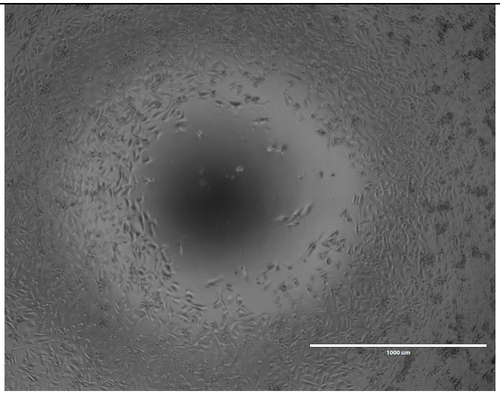 | 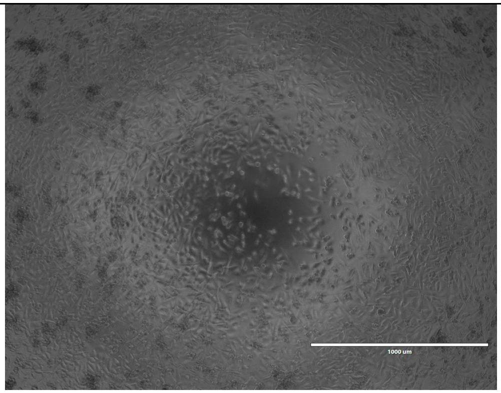 | 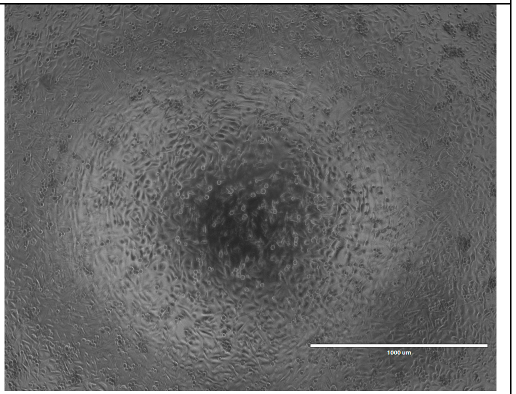 |
| **Paclitaxel** | 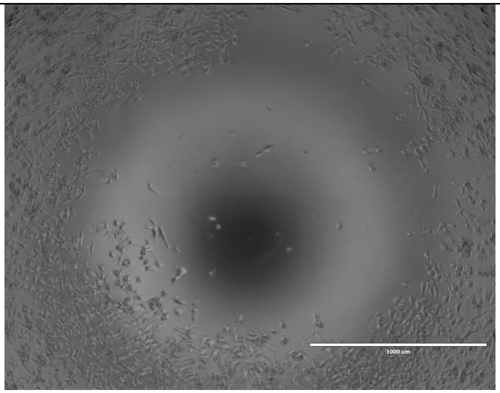 | 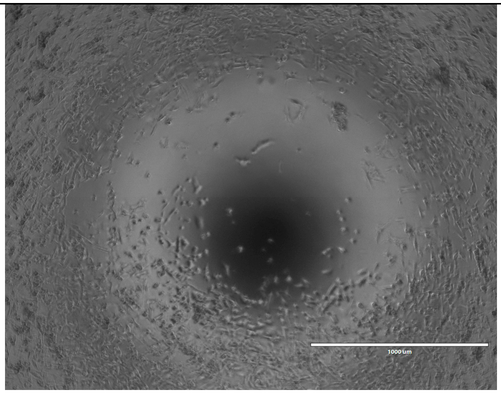 | 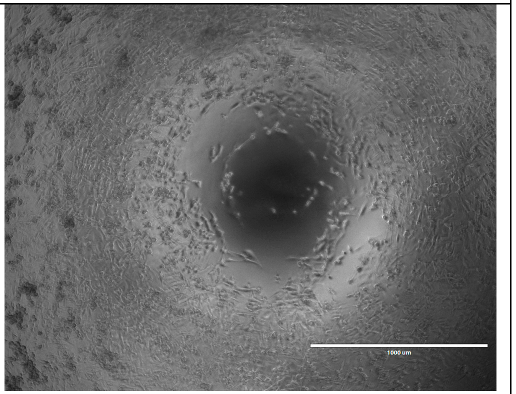 |

1000um

**Figure S7.** Migration of PC3 cells treated with the MNTC of Paclitaxel for 24, 48, and 72 hours viewed under the EVOS® FL Imaging System. Inhibition of migration can be clearly seen after treatment with Paclitaxel compared to the untreated cells. Results shown are representative of three independent experiments.

| Sample/Hour | 24 | 48 | 72 |
|---|---|---|---|
| **MPc** | 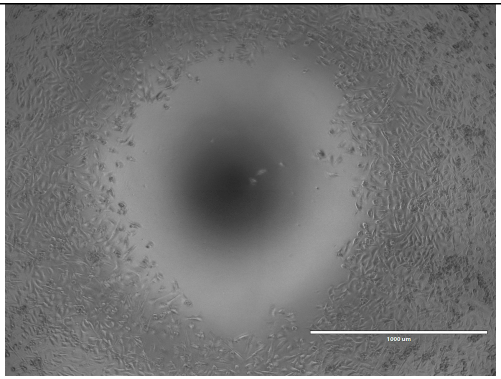 | 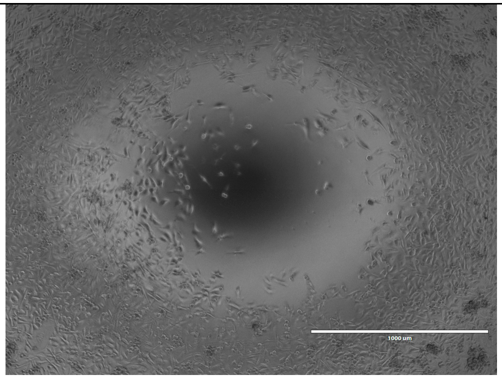 | 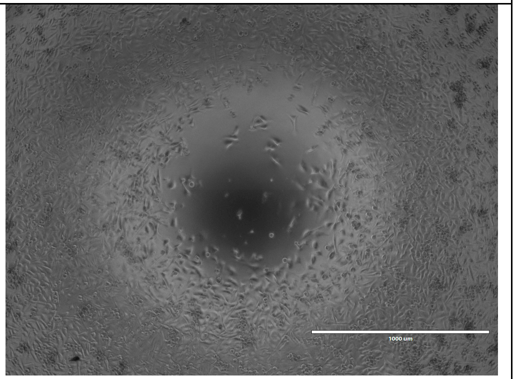 |
| **MPh** | 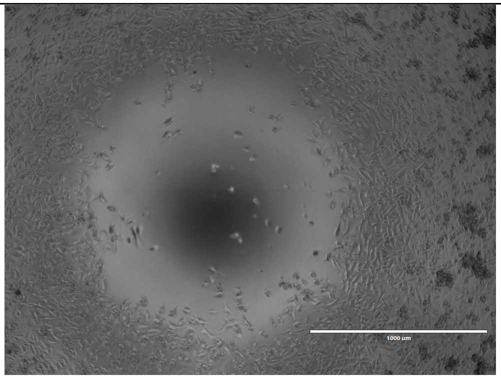 | 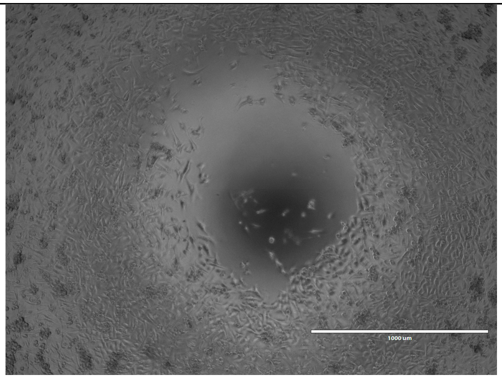 | 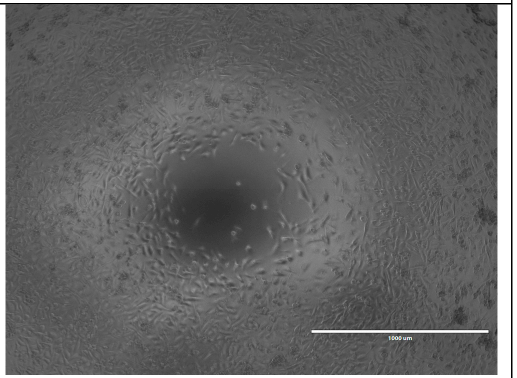 |

**Figure S8.** Migration of PC3 cells treated with the MNTC of the active extracts of *Marantodes pumilum* for 24, 48, and 72 hours viewed under the EVOS® FL Imaging System. Inhibition of migration can be clearly seen after treatment with MPc and MPh compared to the untreated cells. Results shown are representative of three independent experiments.

1000um

| **Sample/Hour** | **24** | **48** | **72** |
|---|---|---|---|
| **FD1c** | 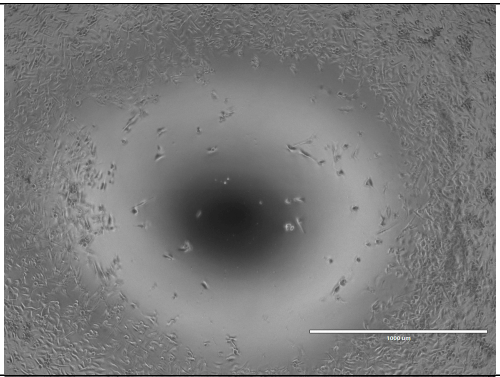 | 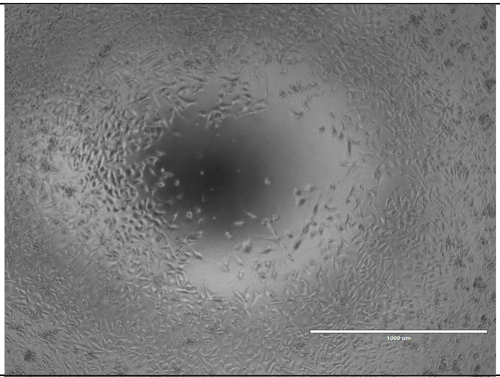 | 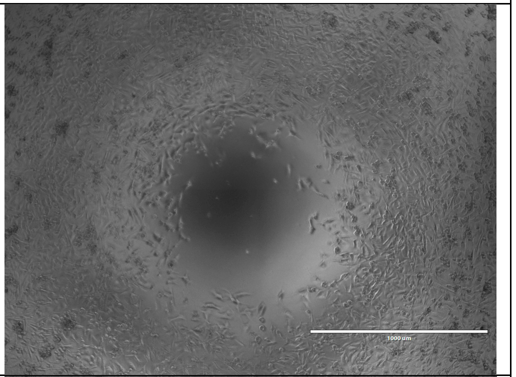 |
| **FD2c** | 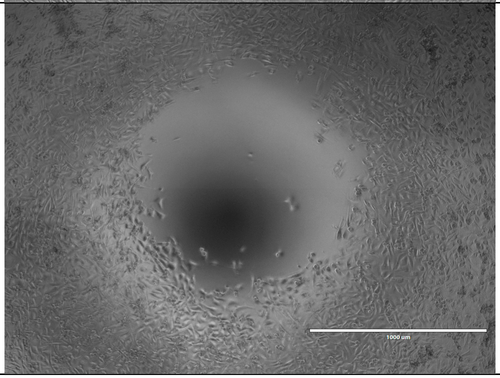 | 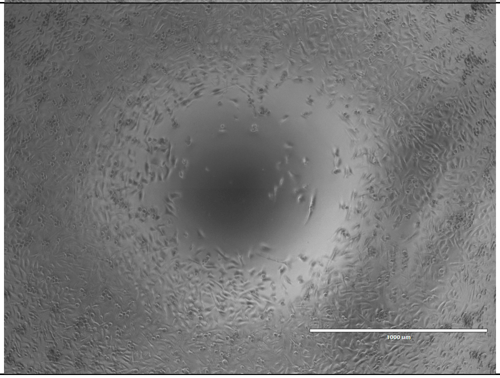 | 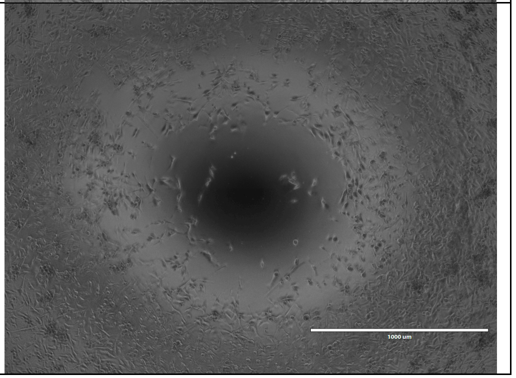 |

1000um

**Figure S9**. Migration of PC3 cells treated with the MNTC of the active extracts of *Ficus deltoidea* for 24, 48, and 72 hours viewed under the EVOS® FL Imaging System. Inhibition of migration can be clearly seen after treatment with FD1c and FD2c compared to the untreated cells. Results shown are representative of three independent experiments.

**4. HPLC-DAD chromatograms of the microfractions**

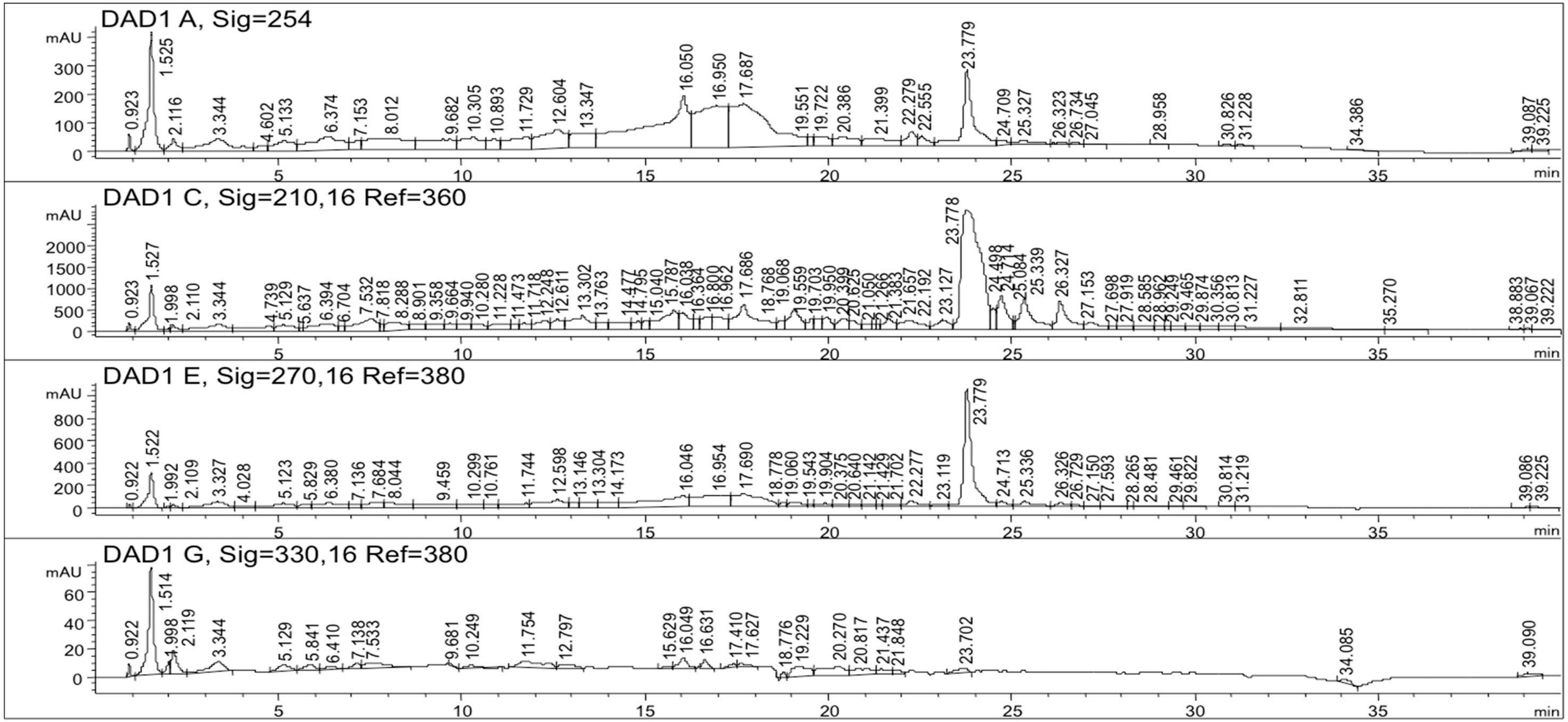


**Figure S10**. HPLC chromatogram of MPc F30-33. 4 different wavelengths were used in this study including 254, 210, 270 and 330nm. The peaks distribution looks almost similar in all investigated wavelengths, however the intensity of each peak is different. Therefore, the scale on the Y-axis is different for each wavelength due to different signal intensities.

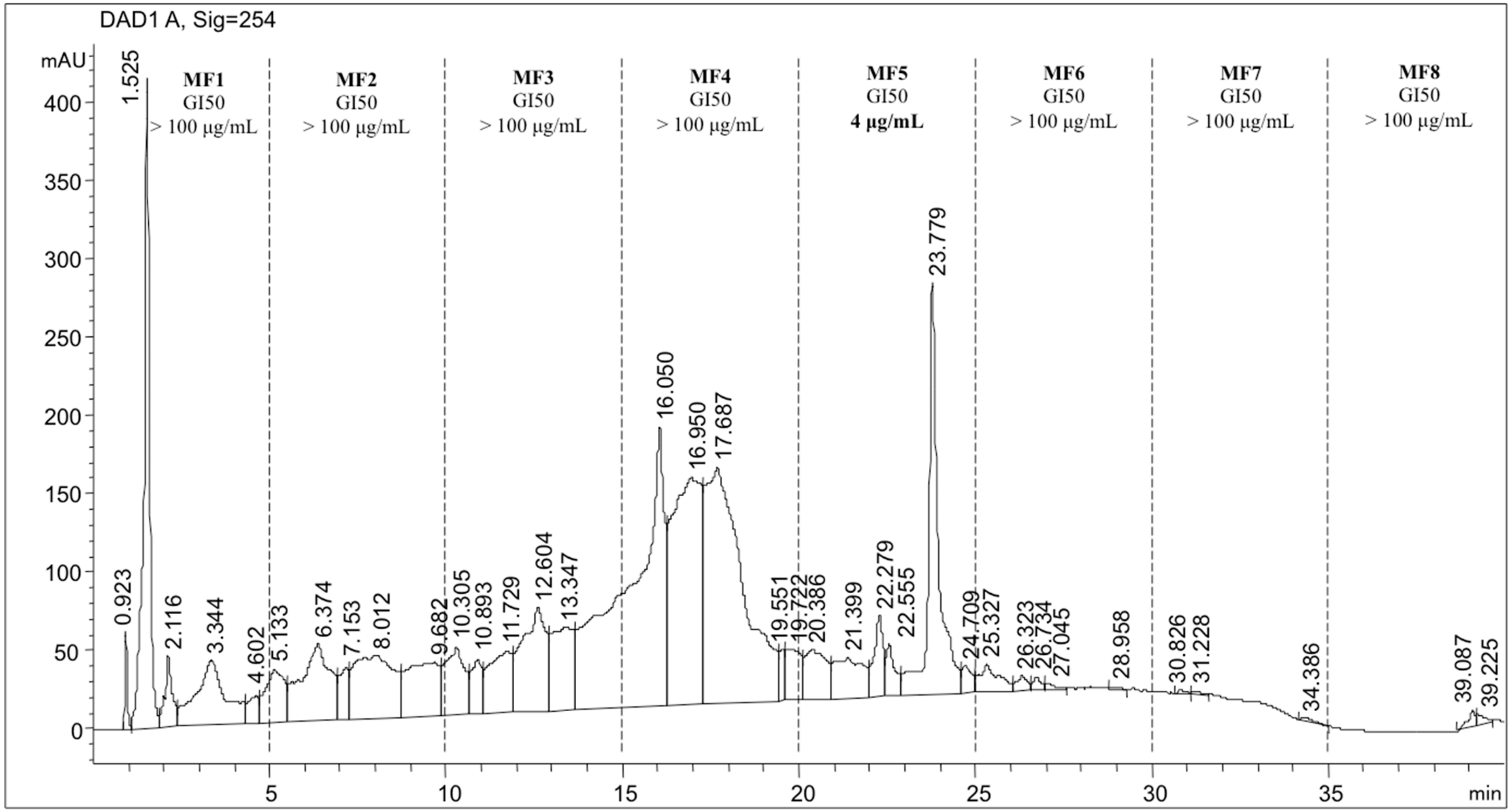


**Figure S11.** Overlay of the HPLC chromatogram of MPc F30-33 with their respective GI50. The GI50 concentrations (µg/mL) of the microfractions determined for PC3 cells as assessed by the SRB assays at 72 hours. Paclitaxel (0.01µM) was used as a reference drug. Each result was obtained in three independent experiments and run in triplicate.

## 5. Spectroscopic analysis of MP-1

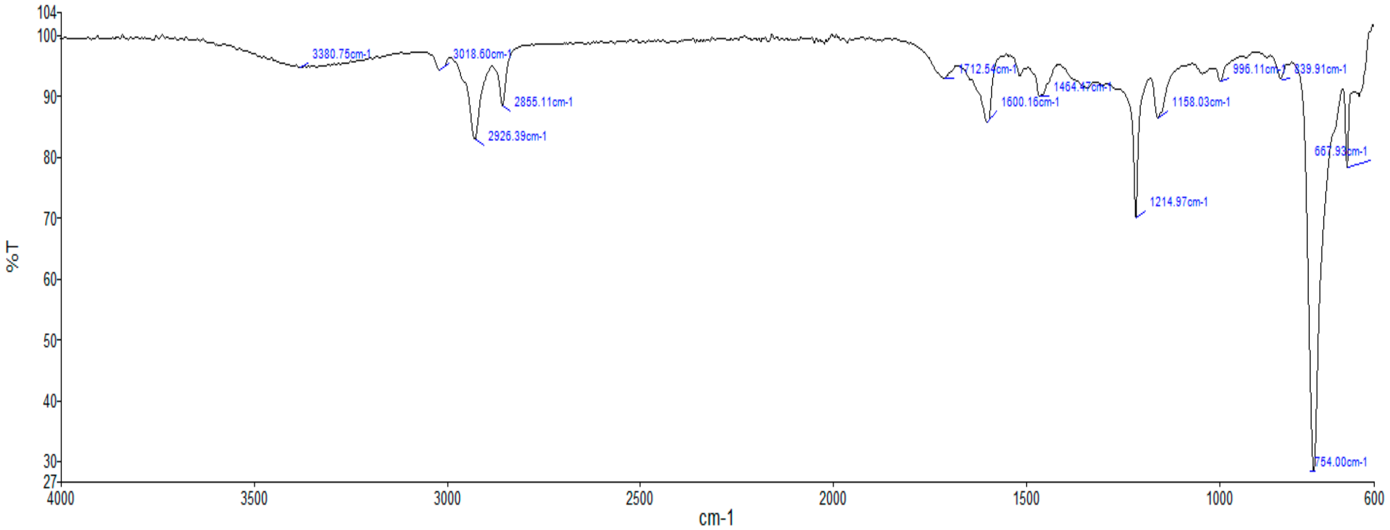


**Figure S12.** Infrared spectra for MP-1

## 6. HR-MS spectra

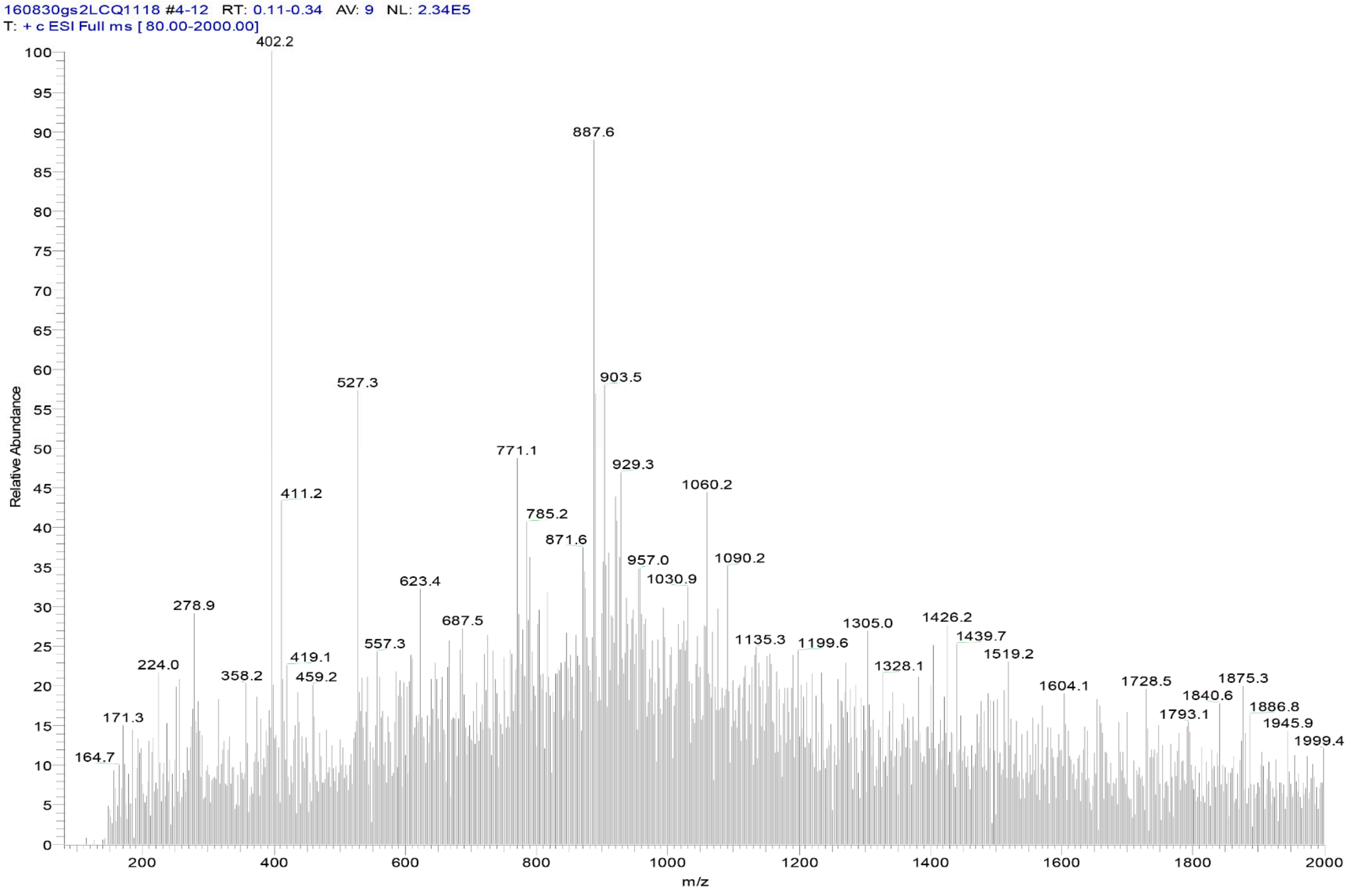


**Figure S13.** MP-1 ESI-MS spectrum

## 7. 1.D and 2D NMR spectra of isolate MP-1.

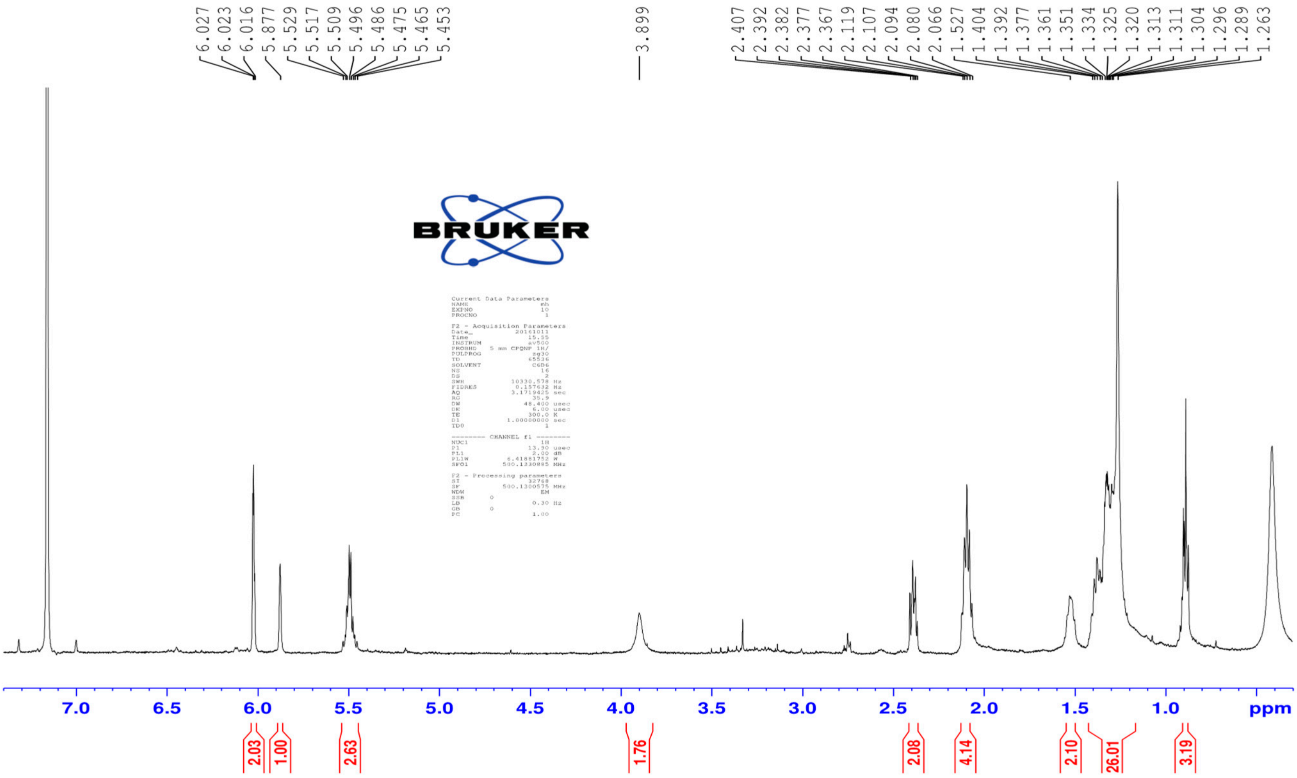


**Figure S14.** 1HNMR of MP-1 dissolved in benzene-*d6* at 500MHz

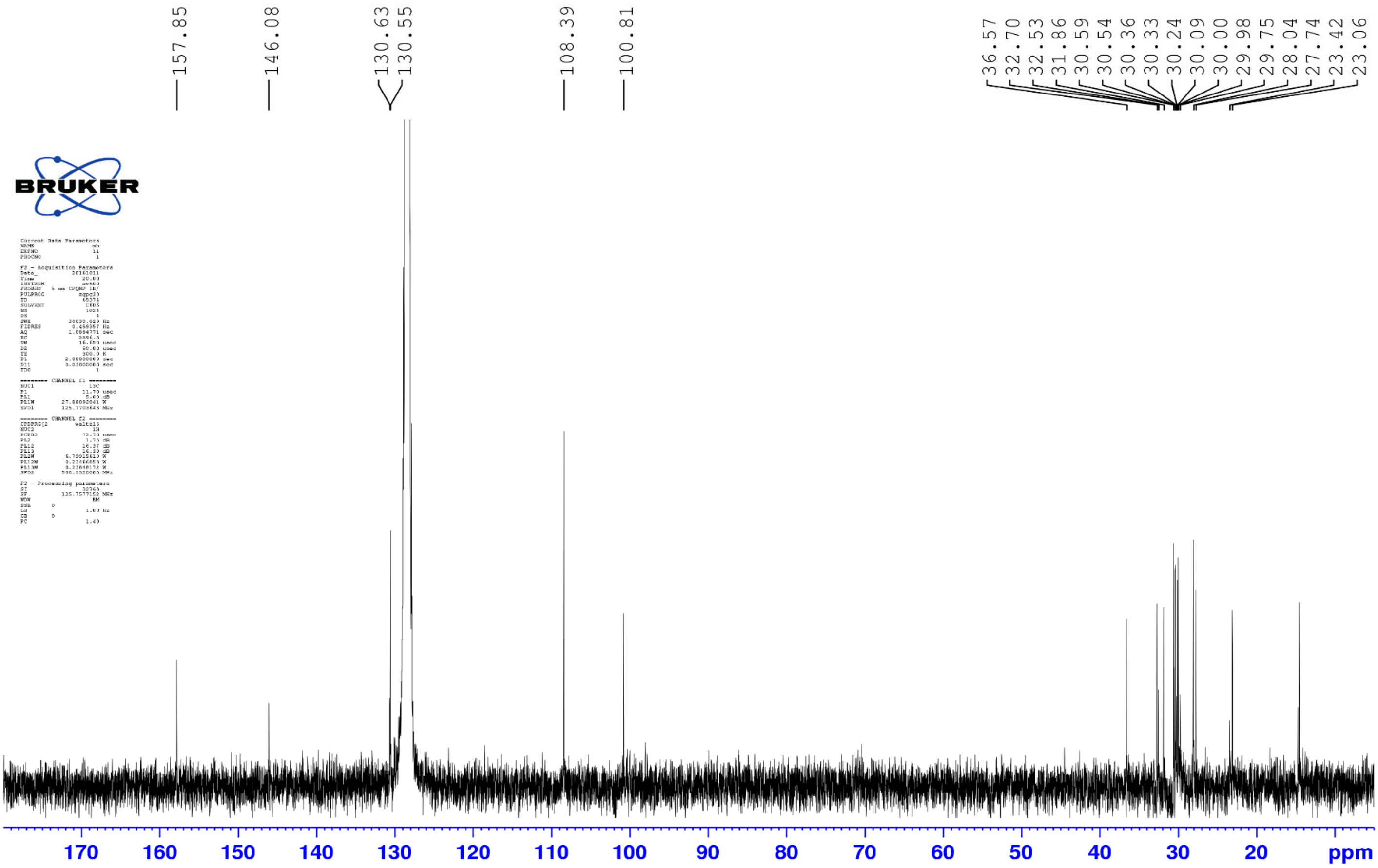


**Figure S15.** 13CNMR of MP-1 dissolved in benzene-*d6* at 500MHz

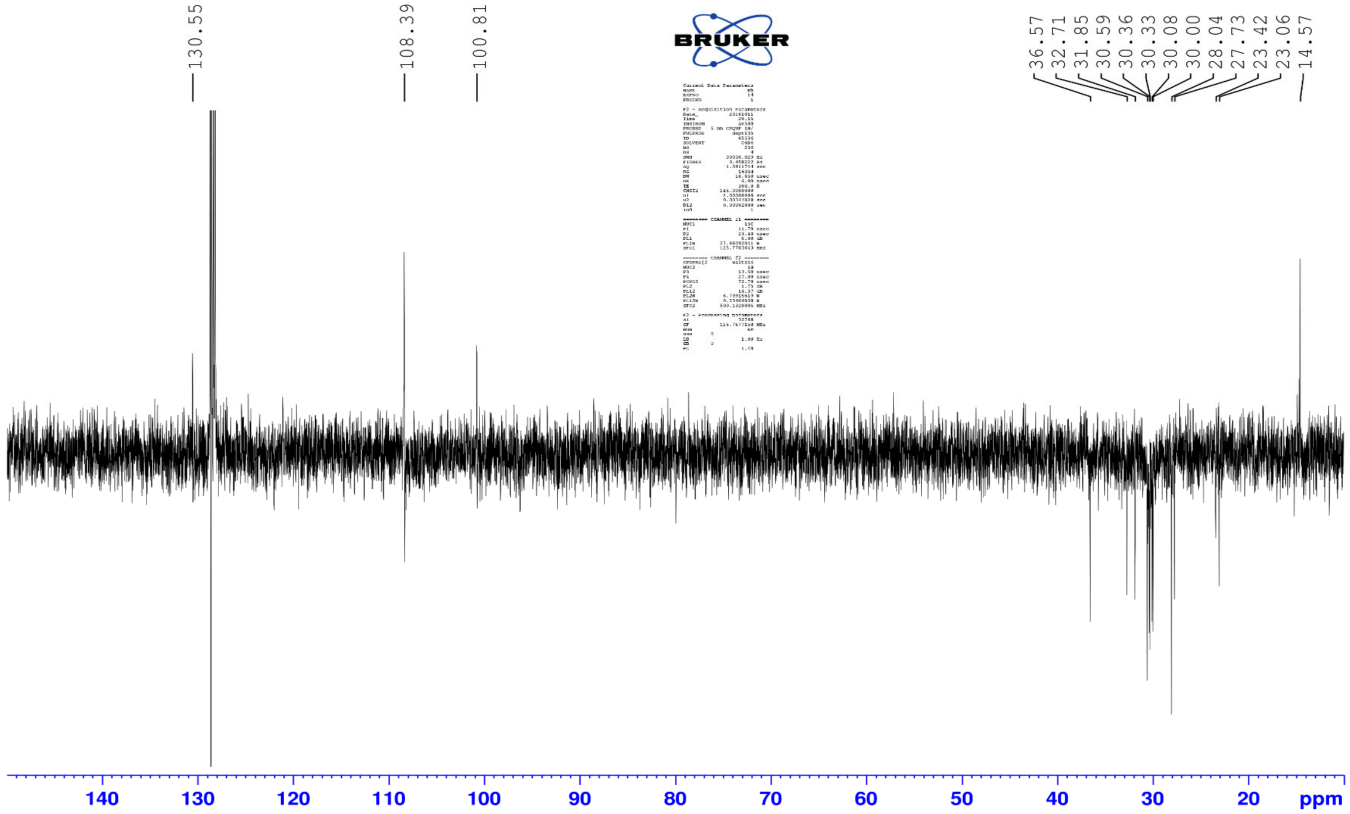


**Figure S16.** DEPT of MP-1 dissolved in benzene-*d6* at 500MHz

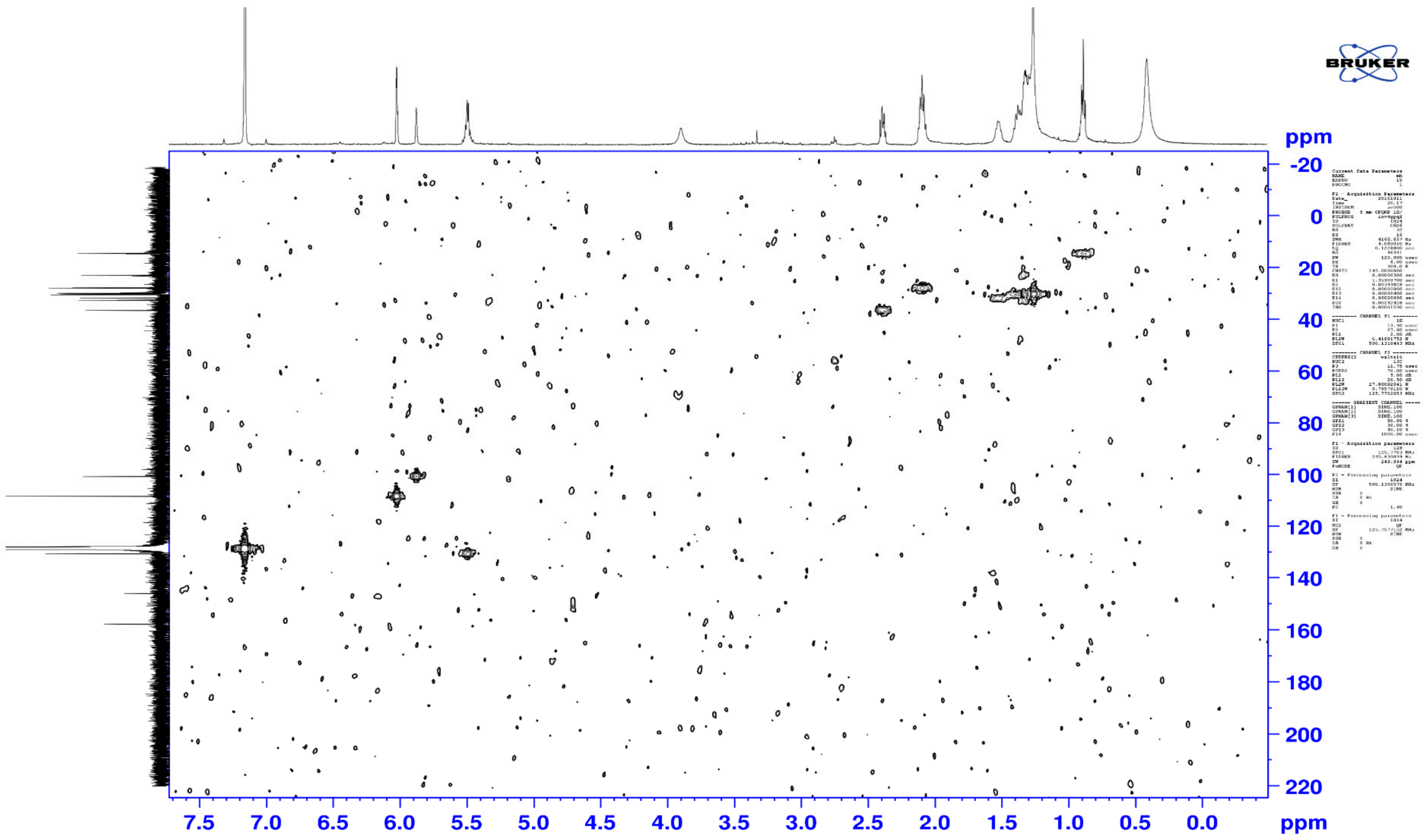


**Figure S17.** HMQC correlations of MP-1 dissolved in benzene-*d6* at 500MHz

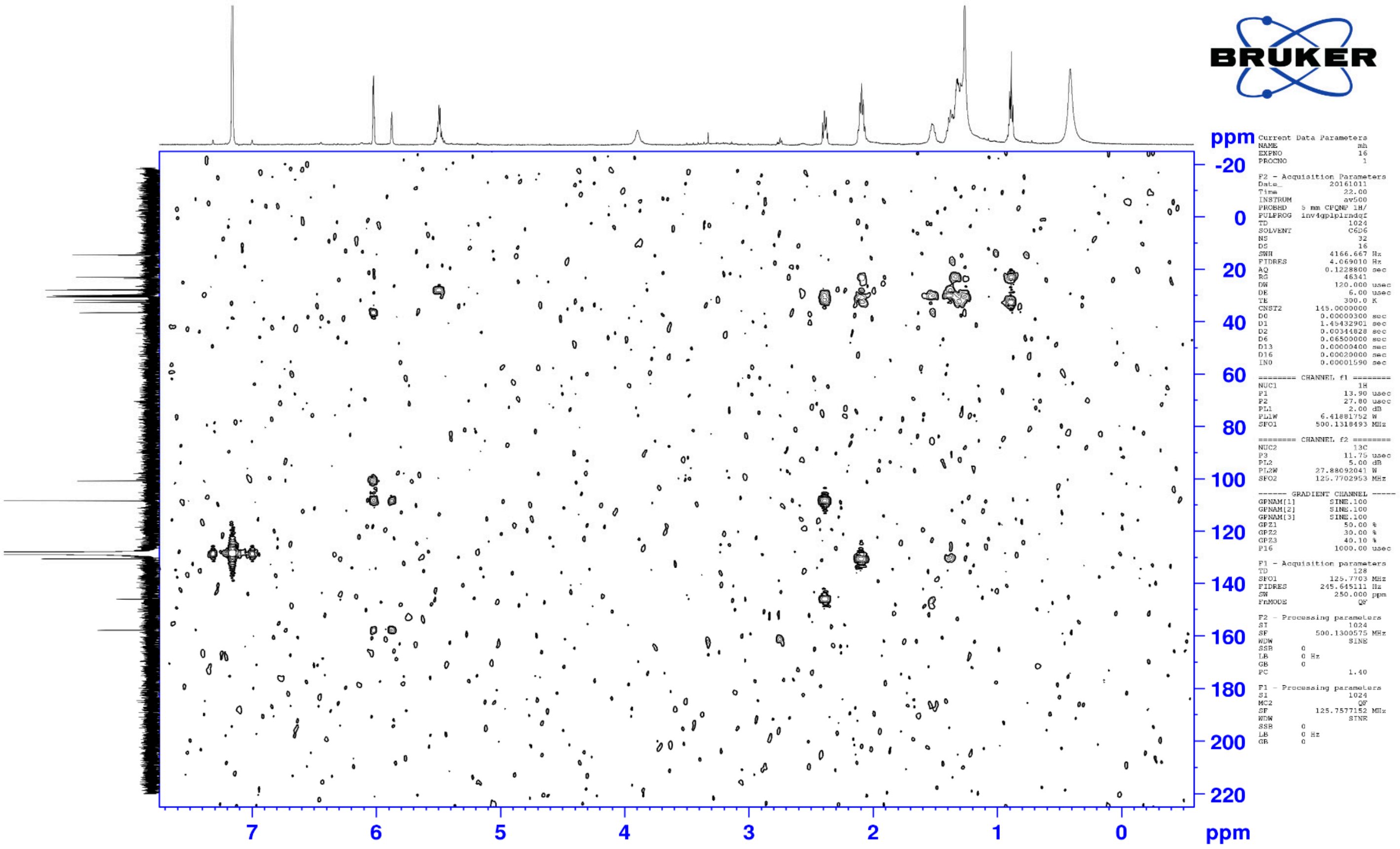


**Figure S18.** HMBC correlations of MP-1 dissolved in benzene-*d6* at 500MHz

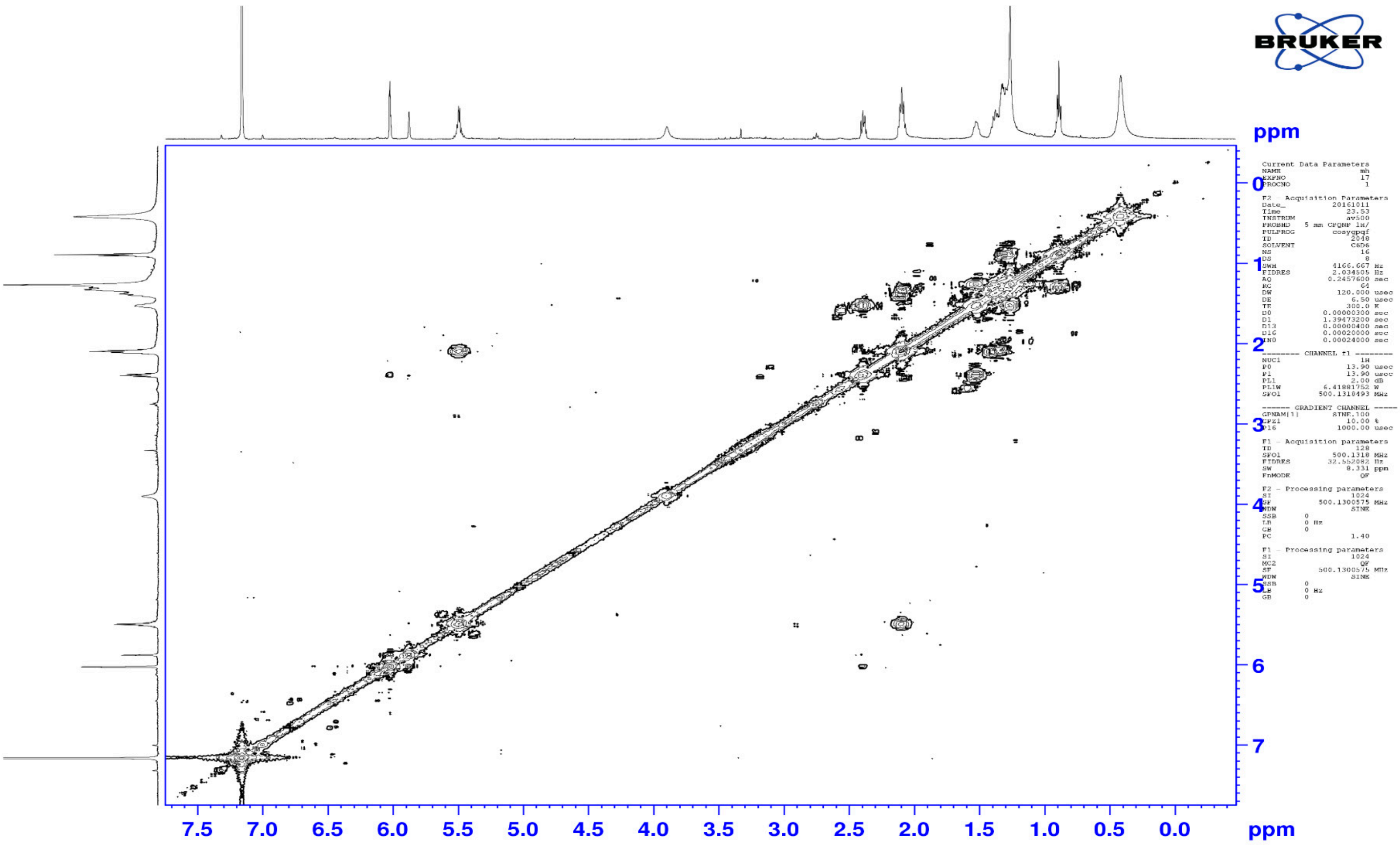


**Figure S19.** COSY of MP-1 dissolved in benzene-*d6* at 500MHz

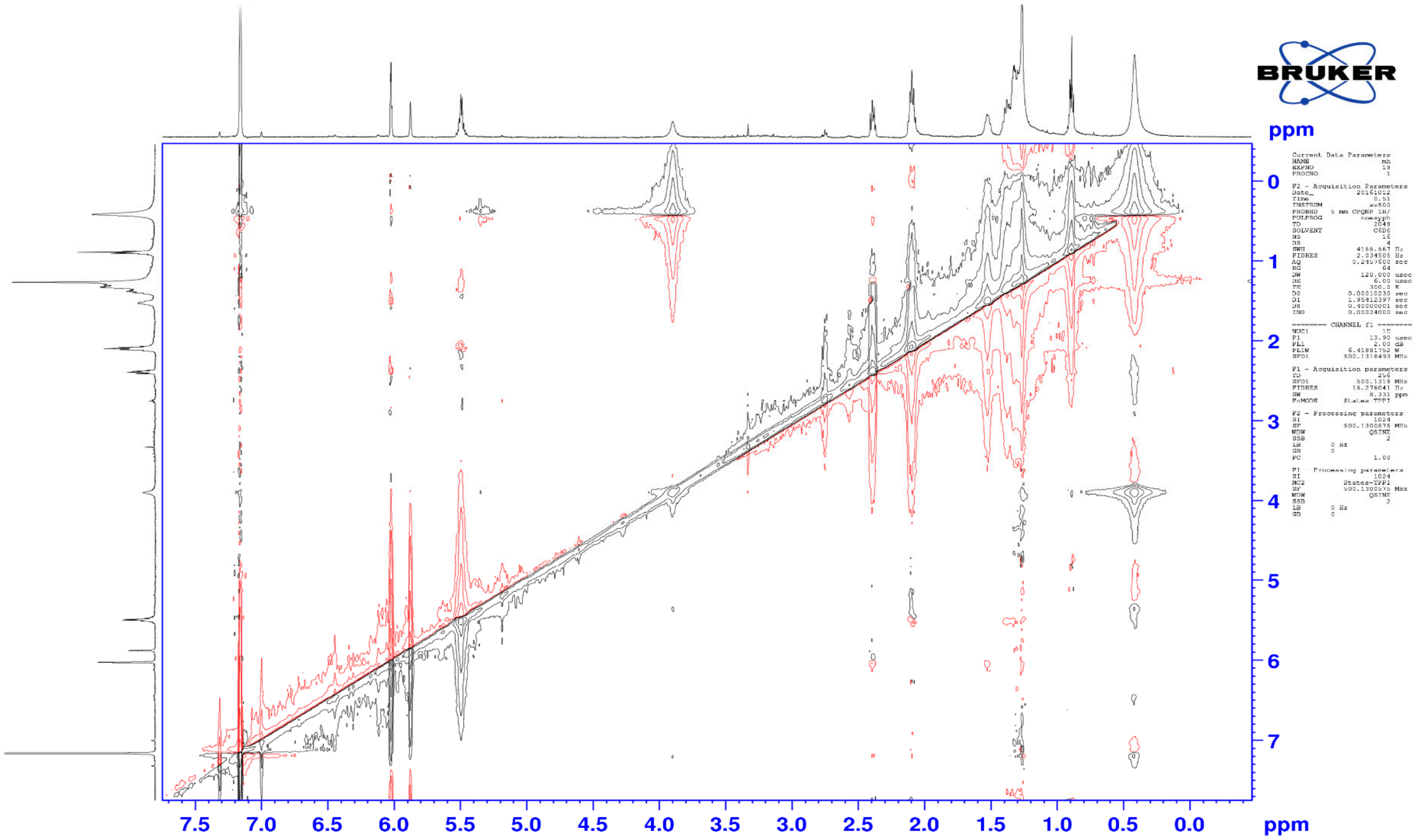


**Figure S20.** NOESY of MP-1 dissolved in benzene-*d6* at 500MHz

**End of Supplementary materials**
